# A tsunamigenic delta collapse and its associated tsunami deposits in and around Lake Sils, Switzerland

**DOI:** 10.1007/s11069-021-04533-y

**Published:** 2021-02-08

**Authors:** Valentin Nigg, Stephan Wohlwend, Michael Hilbe, Benjamin Bellwald, Stefano C. Fabbri, Gregory F. de Souza, Florian Donau, Reto Grischott, Michael Strasser, Flavio S. Anselmetti

**Affiliations:** 1 Institute of Geological Sciences and Oeschger Centre for Climate Change Research, University of Bern, Baltzerstrasse 1+3, 3012 Bern, Switzerland; 2 Geological Institute, ETH Zurich, Sonneggstrasse 5, 8092 Zürich, Switzerland; 3 Volcanic Basin Petroleum Research (VBPR), Høienhald, Blindernveien 5, 0361 Oslo, Norway; 4 Institute of Geochemistry and Petrology, ETH Zurich, Clausiusstrasse 25, 8092 Zürich, Switzerland; 5 Department of Geology, University of Innsbruck, Innrain 52, 6020 Innsbruck, Austria

**Keywords:** Delta collapse, Tsunami deposit, Lacustrine tsunami, Tsunami modeling, Sedimentology

## Abstract

Large lacustrine mass movements and delta collapses are increasingly being considered as potential tsunamigenic sources and therefore hazardous for the population and infrastructure along lakeshores. Although historical reports document tsunami events in several lakes in Switzerland, and although the propagation of lake tsunamis has been studied by numerical wave modeling, only little is known about on- and offshore lacustrine tsunami deposits. In Lake Sils, Switzerland, a large prehistoric mass-movement deposit originating from the Isola Delta with a minimum estimated volume of 6.5 × 10^6^ m^3^ and a basinal thickness of > 6 m in the seismic record has been identified by previous studies and radiocarbon dated to around 700 Common Era. Here, we combine (i) comprehensive sedimentological investigation of sediment cores recovered from the on- and offshore settings, (ii) mineralogical fingerprinting of the inflows from key catchments to characterize sediment provenance, and (iii) numerical tsunami modeling, to test the hypothesis of a tsunamigenic delta collapse in Lake Sils. We observe a clastic event deposit consisting of coarse-grained, fining-upward sand overlying an organic-rich peat deposit in the shallow water. This layer thins and fines landward on the coastal plain. Toward the deeper water (20–40 m), the deposit transforms into a thicker and more heterogeneous sediment package with multiple sequences of fining-upward sand and a well-pronounced clay cap at the top. Radiocarbon dating of the peat underlying the event deposit yields a maximum age of 225–419 calibrated  Common Era. The tsunami models, which indicate wave heights reaching up to 5 m, simulate areas of inundation that coincide with the location of event deposits. Based on our results, we propose that the historically undocumented Isola Delta collapse generated a basin-wide tsunami that inundated the lakeshore, transporting large amounts of unconsolidated sediment along the lakeshore toward the coastal plain and into the deeper lake basin.

**Supplementary Information:**

The online version of this article (10.1007/s11069-021-04533-y) contains supplementary material, which is available to authorized users.

## Introduction

Historical documents and recent scientific investigations provide evidence that large subaqueous mass movements and delta collapses are capable of generating tsunamis in lakes (Hilbe and Anselmetti 2015; Kremer et al. 2012). Besides impacts from rockfalls and subaerial landslides, subaqueous mass movements are considered as the most common triggering mechanism for lacustrine tsunami generation. Even though their wavelength is much shorter than that of their marine counterparts, lacustrine tsunamis have wavelengths of several hundred meters, which clearly distinguish them from wind-induced waves in these basins. At Lake Lucerne in central Switzerland, historical documents report anomalously large waves in 1601 and 1687 (Hilbe and Anselmetti 2014). These effects were attributed to large subaqueous mass movements, which were adequately simulated with numerical tsunami generation and propagation models (Hilbe and Anselmetti 2015). Other historical documents report a severe tsunami in Lake Geneva generated by the Rhone Delta collapse (Kremer et al. 2012). In Lake Brienz, a small-scale tsunami was observed following a partial collapse of the main delta in 1996 (Girardclos et al. 2007).

Although tsunamis are reported in lakes, the related on- and nearshore lacustrine tsunami deposits are rarely documented. For example, subaqueously generated boulder ridges, sediment-wave channels, and gently sloping tsunami erosion surfaces provide morphological evidence for a prehistoric tsunami in Lake Tahoe (Nevada-California, USA; Moore et al. (2014)). In the shallow Lake Owens (California, USA), unusual poorly sorted and upward-graded pebbly sand with mud is likely related to the 1872 earthquake-induced seiche (wave height ~ 50 cm) that eroded much of the lakebed (Smoot et al. 2000). In Lake Patzcuaro (Mexico), heterogeneous sand and silt with angular lithoclasts, ceramic artifacts and abundant remains of fish bones, bivalves, gastropods and pelagic species are deposited above an erosional unconformity to the underlying unit (Garduño-Monroy et al. 2011). In Lake Chehalis (Canada), a subaerial landslide-generated tsunami-induced severe shore destruction in 2007 (Roberts et al. 2013).

In marine settings, on the other hand, tsunami deposits are widely studied to infer past tsunami events in a broad range of coastal areas (e.g., Bourgeois et al. 2009; Costa and Andrade 2020; Dawson and Stewart 2007; Engel and Brückner 2011). During tsunami inundation and backwash, a vast amount of shoreface and beach sediment is eroded, transported, and redeposited in the coastal environment, and subsequently moved offshore (e.g., Einsele et al. 1996; Fujiwara and Kamataki 2007; Goto et al. 2011; Paris et al. 2010; Sakuna et al. 2012; Sugawara et al. 2008). The associated tsunami deposits are commonly site specific and characterized by a wide range of sedimentological features depending on coastal geomorphology and microtopography (e.g., Hori et al. 2007; Matsumoto et al. 2016; Nishimura et al. 2015), sediment availability (e.g., Dawson and Shi 2000; Goff et al. 2009; Meilianda et al. 2010), as well as tsunami magnitude (e.g., Yamaguchi and Sekiguchi 2015; Ishimura and Yamada 2019; Putra et al. 2019), and preservation potential (e.g., Brill et al. 2020; Goto et al. 2012; Spiske et al. 2020; Szczuciński 2012). Hence, research on tsunami deposits requires multiproxy sedimentary analyses, including sedimentological, geochemical, and biological approaches (e.g., Goff et al. 2010; Judd et al. 2017; Ramirez-Herrera et al. 2012). Sedimentary structures such as erosional basal contacts, fining-upward sequences, and rip-up clasts are the most common physical textural characteristics found in onshore tsunami deposits in various coastal settings worldwide (e.g., Bondevik et al. 2005; Gelfenbaum and Jaffe 2003; Srinivasalu et al. 2009). On the other hand, tsunami events may leave no traces in the geological record, especially along rocky coasts, where sediment supply is limited (e.g., Dawson and Shi 2000), or tsunami inundation may be indicated through erosional unconformities in coastal sand barriers (e.g., Costa et al. 2016). Additionally, geochemical proxies such as Na, S, and Cl concentrations (e.g., Goff et al. 2012; Szczuciński et al. 2006) and/or biogenic contents are used to identify marine tsunami deposits; for example, pelagic and benthic fauna are used to infer tsunami deposits and their sediment source (e.g., Garrett et al. 2015; Kitamura et al. 2018; Smedile et al. 2020; Szczuciński et al. 2012; Tanigawa et al. 2018). Terrestrial sedimentary records are used to infer minimum tsunami inundation (e.g., Chagué-Goff et al. 2012; Moreira et al. 2017) and run-up height (e.g., Bondevik et al. 2005; Costa et al. 2016; La Selle et al. 2020; Paris et al. 2020). Based on the internal structure, composition, and spatial distribution of tsunami deposits, it may be possible to estimate magnitude and flow conditions by tsunami inverse modeling (e.g., Jaffe and Gelfenbaum 2007; Jaffe et al. 2012; Spiske et al. 2010; Woodruff et al. 2008). Such studies are directly applicable for coastal tsunami hazard assessments (e.g., Engel et al. 2016; Leonard et al. 2014 and references therein).

Compared to the onshore realm, the number of scientific publications describing offshore tsunami deposits is limited (e.g., Dawson and Stewart 2008). Although the combined investigation of on- and offshore tsunami deposits may provide a more robust and accurate reconstruction of past events (Costa and Andrade 2020), only few case studies describe offshore tsunami deposits (e.g., Goodman-Tchernov et al. 2009; Paris et al. 2010; Smedile et al. 2020; Tamura et al. 2015). For example, Sakuna et al. (2012) describe poorly sorted mud including terrigenous and anthropogenic components, which were transported from backwash currents of the 2004 Indian Ocean tsunami into the shallow marine environment of the Andaman Sea off the coast of Thailand. Paris et al. (2010) characterize tsunami-derived boulder deposits from the 2004 Indian Ocean tsunami in the offshore setting Lhok Nga, Indonesia. Recently, tsunami backwash deposits from the 2009 South Pacific tsunami and 1960 Great Chilean earthquake tsunami were encountered in sediment cores from the sheltered Pago Pago Bay (USA) based on grain-size analysis, geochemical proxy analysis, sediment thin sections, and ^137^Cs and ^210^Pb dating (Riou et al. 2020). These deposits are characterized by terrigenous sediment transported as a dense and cohesive hyperpycnal flow that induced shearing of the underlying sediment (Riou et al. 2020).

**Fig. 1:**
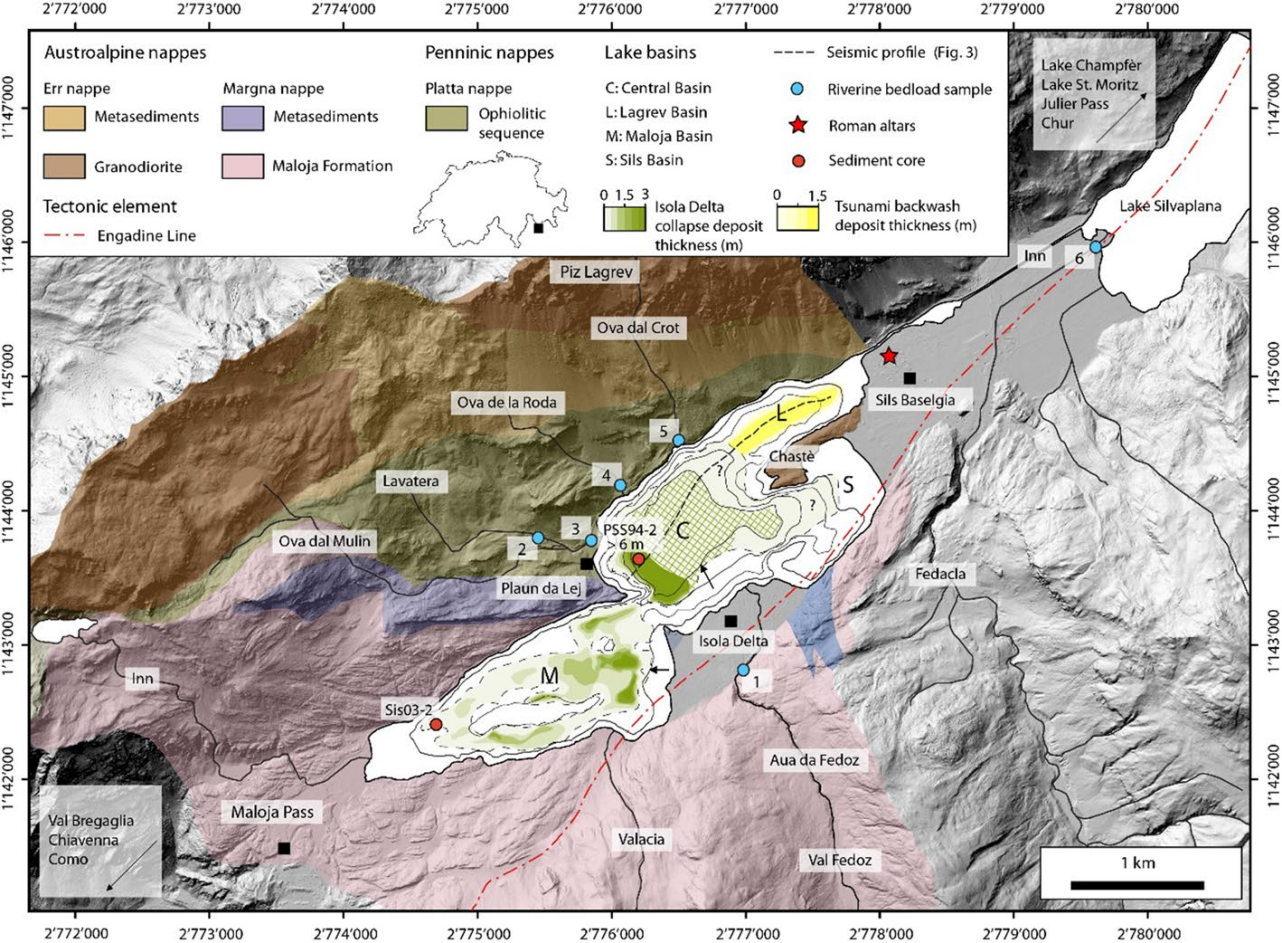
Hillshade map showing Lake Sils and its catchment geology (swissALTI3D, swisstopo). The major tectonic and geological units are modified after Spillmann and Büchi (1993) and 1:25′000 Swiss geological map (GA24, swisstopo). Note the Engadine Line that runs along the southern shore of the lake. Lake Sils’ four sub-basins are indicated (*M* Maloja Basin, *C* Central Basin; *L* Lagrev Basin and *S* Sils Basin). The Isola Delta collapse mass-movement deposit is highlighted in light green. Hashed green areas indicate area where thickness is not resolved by reflection seismic data due to limited penetration, but where the deposits is likely thicker than 2.5 m. Location of riverine bedload samples is indicated with numbered blue dots (1: Aua da Fedoz; 2: Lavatera 3: Lavatera + Ova dal Mulin; 4: Ova de la Roda; 5: Ova dal Crot; 6: Fedacla)

Based on sediment cores collected on the coastal plain and in the shallow water, this study provides evidence for a lacustrine tsunami event in the proglacial Lake Sils, located in the Upper Engadine, Switzerland (Fig. 1). The proposed mechanism for the tsunami initiation is a large delta-slope collapse around 548–797 calibrated Common Era (cal CE) with an estimated minimum volume of the mass-movement deposit in Lake Sils of 6.5 × 10^6^ m^3^ (Blass et al. 2005).

## Study site

Lake Sils (surface area: 4.1 km^2^) is located in the Upper Engadine in southeastern Switzerland at 1797 m above sea level (m a.s.l). The lake is connected downstream with Lake Silvaplana, Lake Champfèr, and Lake St. Moritz, draining the valley toward the northeast (Fig. 1). The Maloja Pass (1815 m a.s.l) in the southwest of Lake Sils separates the Engadine valley from the Val Bregaglia, which drains southward toward Chiavenna in northern Italy. The catchment of Lake Sils is situated in a complex geological area consisting of the Austroalpine and Penninic nappes (Fig. 1). The Austroalpine nappes consist of the Margna nappe to the south of the lake and the Err nappe north of the lake, respectively (Spillmann and Büchi 1993). The Penninic Platta nappe in the northeast consists of an ophiolitic sequence originating from the South Penninic realm (Dietrich 1970). A major regional tectonic element is the Engadine Line, an oblique sinistral strike-slip fault (Trümpy 1977), which runs along the southeastern part of Lake Sils. This fault can be traced from Lake Sils 30 km toward the northeast and 25 km in southwest direction (Tibaldi and Pasquarè 2008).

The major incoming tributary, the Aua da Fedoz, originates in the Val Fedoz and feeds the Isola Delta, the main delta of the lake (Fig. 1). The Ova dal Mulin, Ova de la Roda, Ova dal Crot, and Lavatera are minor tributaries draining from the northwest, and the Inn and the Valacia from the west and south, respectively. The Fedacla River currently feeds into Lake Silvaplana, but also fed into Lake Sils at least temporarily during high-discharge events (Ohlendorf 1998). Lake Sils’ four sub-basins form a longitudinal lake morphology along the main valley axis. The Central Basin is the deepest and reaches a depth of 72 m. The Lagrev Basin in the northeast is separated from the Sils Basin by the Chastè, a peninsula with outcropping bedrock composed of mylonitic granodiorites belonging to the Maloja Formation (Fig. 1). The Maloja Basin reaches a depth of 30 m and forms the southwestern part of the lake. Unlike delta areas, such as the Isola Delta on the southern shore, where sediments are dominated by coarse clastic sediments intercalated by few peaty horizons (Grischott et al. 2017), the coastal plain at Sils Baselgia is characterized by organic-rich swampy deposits onshore, fine clastic sediments, and lake-derived organics, which are occasionally interrupted by coarser clastic layers.

Pollen assemblages from sediment cores taken in Lake Champfèr and Lake St. Moritz provide evidence for first human impact in the Upper Engadine during the Neolithic Period around 5500 cal Before Present (BP) (Gobet et al. 2003). However, archeological findings are rather sparse for that time period in the area (Nauli 1981; Rageth 2000). Marked vegetation changes and regular cereal cultivation started around 3900 cal BP (Gobet et al. 2003). A late Bronze Age spring tapping, which is the only well-preserved wood building of the Swiss Alpine prehistory, was built at St. Moritz in 3361 cal BP (Oberhänsli et al. 2015). Although no permanent settlements are documented during that time, mule tracks were frequently used along the main passes in the Alpine environment as well as in the Upper Engadine (Roth-Bianchi 2007). During Roman times, the area of Lake Sils hosted an important traffic axis (Oberhänsli et al. 2015; Rageth 2004). The directory of the most important Roman roads, the Itinerarium Antonini (230 CE), and the Tabula Peutingeriana (364 CE), an illustration of the Roman road network, report two main road connections in the area from Como in northern Italy to Chur in southeastern Switzerland. One of the two routes crossed the Upper Engadine from Chiavenna to Chur via the Maloja and Julier Passes, being the only route that was passable with two-wheeled carts in the province Raetia prima (Rageth 2004; Roth-Bianchi 2007). With the withdrawal of Roman troops from the province Raetia prima in 401 CE, the Roman presence in the area ended (Ducrey 2006). Consequently, the use of the pass roads and mule tracks in the area decreased in the following centuries (Roth-Bianchi 2007).

**Fig. 2:**
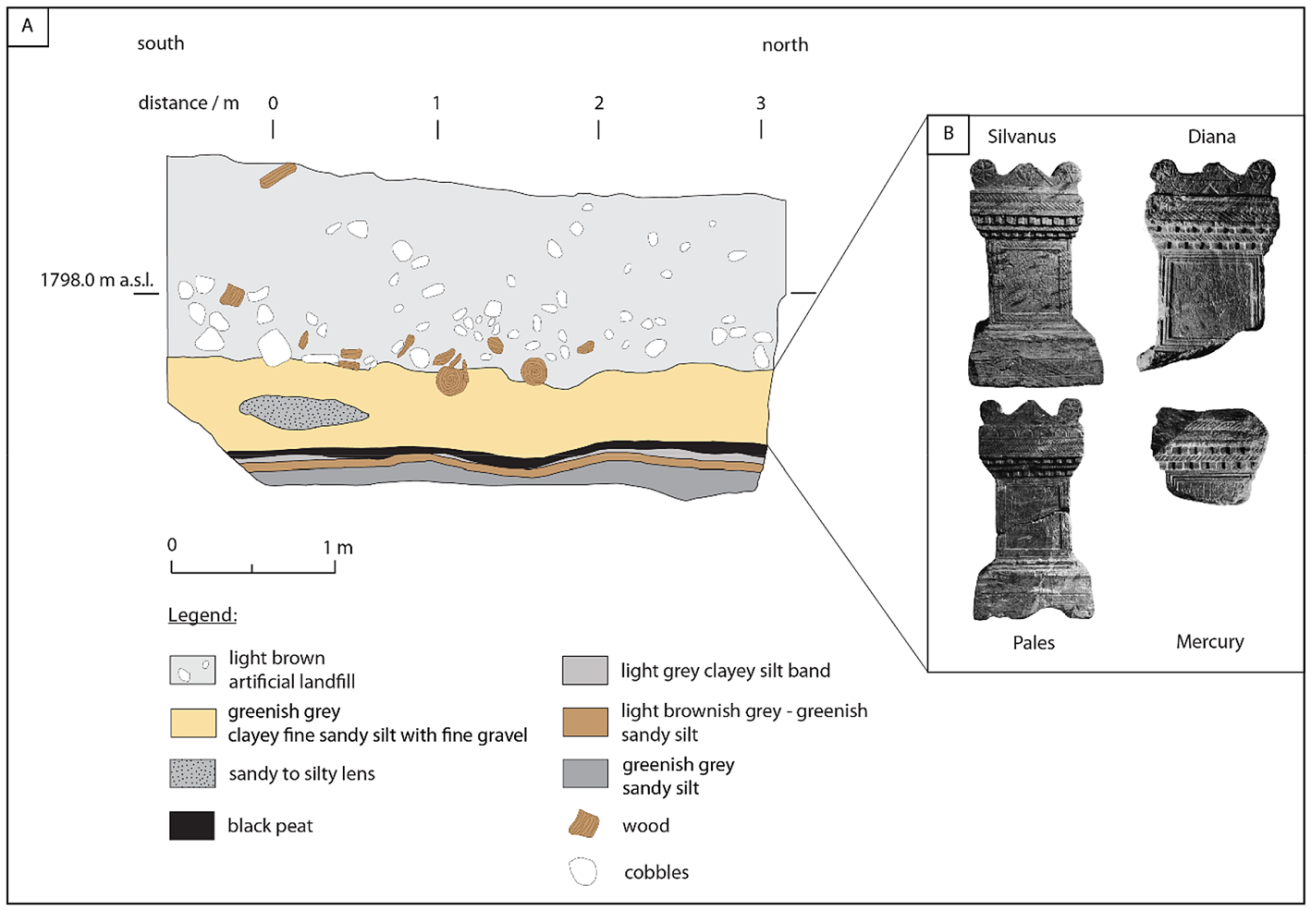
**a** Sketch of sedimentary section through an excavation site at Sils Baselgia (no vertical exaggeration; modified after Rageth (2002)). A peat horizon (black) with a fine band of clayey silt is overlain by a gray to greenish clayey fine sandy silt with fine gravel deposit (orange). It is supposed that the Roman altars (Fig. 2b) were buried in the equivalent unit 30 m away (Rageth 2002). **b** Photographs of the Roman altars that are dedicated to the Roman tutelary deity Silvanus, Diana, Pales and Mercury and excavated at Sils Baselgia in 1964 (Photographs: Archeological Service of Canton Grisons)

Initially, this study was stimulated by archeological findings in Sils Baselgia (Fig. 2), located at the northeastern shore of Lake Sils. During construction work in 1964, four Roman votive altars were found (Erb et al. 1966). The excavated sacrificial altars are 40–47 cm high, made of serpentinite and dedicated to the Roman gods Silvanus, Diana, Pales and Mercury (Fig. 2b). These altars were found 2 m below today’s surface, embedded in a clayey fine sandy silt with fine gravel (Erb et al. 1966). At the time of the discovery, the deposit was interpreted as a lacustrine deposit (Erb et al. 1966). Therefore, the archeologists proposed that the Roman altars fell from a ship during a phase of lake-level high stand connecting Lake Sils with Lake Silvaplana to the northeast (Erb et al. 1966). However, we are not aware of another study supporting the hypothesis of a significantly higher lake level during the Roman era. The fact would further imply massive hydrological changes along the Engadine valley. Based on our findings presented in this study, we propose an alternative hypothesis than regular lake sediments embedding the altars. We present evidence that the Isola Delta collapse generated a basin-wide tsunami that inundated the coastal plains around Lake Sils and partly buried the Roman altars with remobilized sediment around 548–797 cal CE.

### The Isola Delta collapse in previous studies

**Fig. 3:**
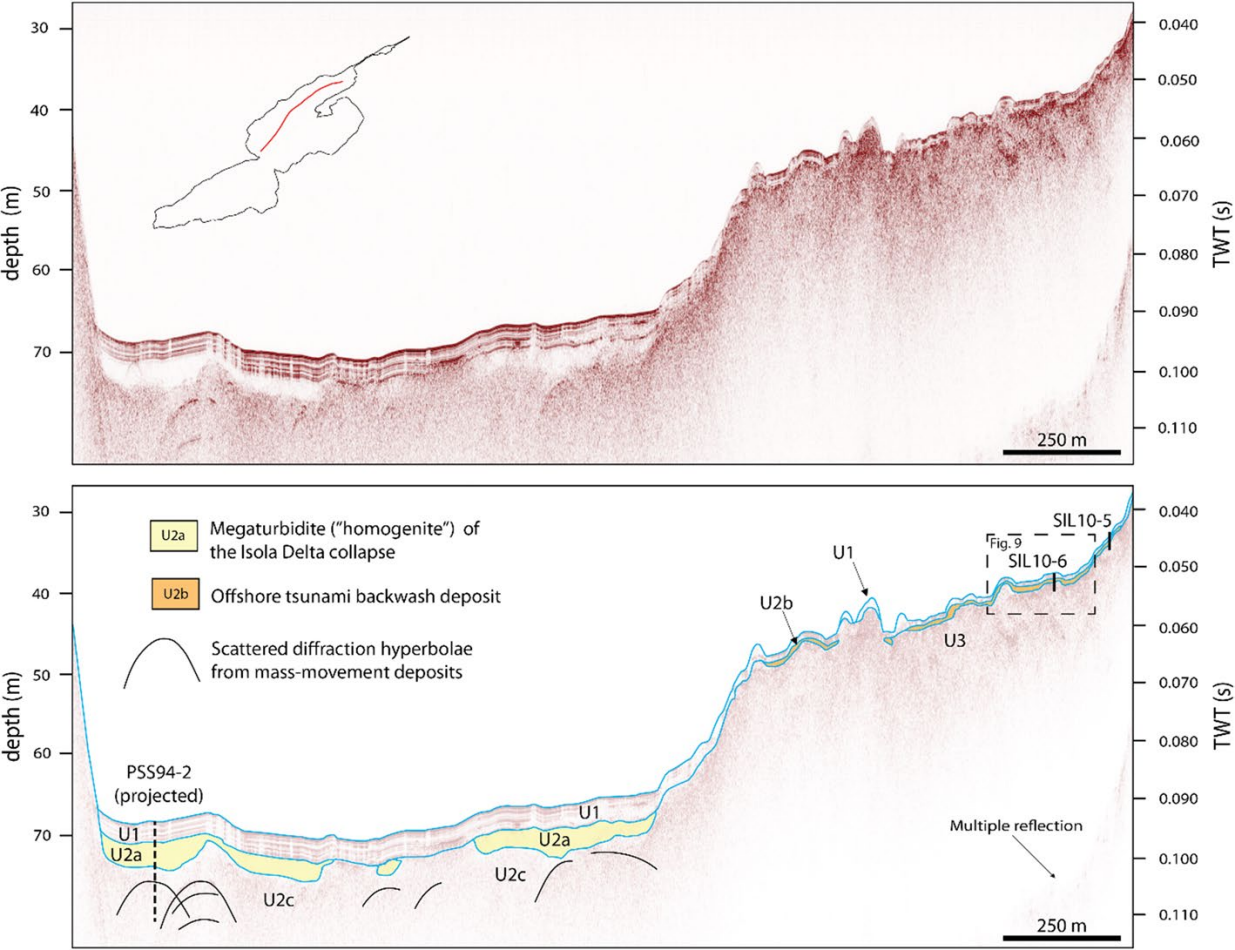
Non-interpreted (top) and interpreted (bottom) seismic reflection profiles along the Central and Lagrev Basins, imaged by a 3.5 kHz single-channel pinger system (vertical scales: two-way travel time (TWT) in seconds (right) and depth in meters with a constant p-wave velocity of 1500 m s^−1^ applied for time-to-depth conversion (left)). Unit 1 (U1) is characterized by continuous high-amplitude reflections with draping character. The underlying Subunit 2a (U2a) represents a homogeneous mud of the Isola Delta collapse mass-movement deposit (Blass et al. 2015). The underlying chaotic Subunit 2c (U2c) represents the lower part of the Isola Delta collapse mass-movement deposit. Subunit 2b (U2b) and Unit 3 (U3) are solely found along the Lagrev Basin (see Fig. 9 for close-up view). Location of the recovered sediment Cores SIL10-5 and SIL10-6 is projected onto the seismic reflection profile modified from Blass et al. (2005)

High-resolution, single-channel seismic data from Lake Sils revealed two seismic units in the shallow subsurface that can be distinguished in the Maloja and Central Basins (Blass et al. 2005). The upper seismic unit is characterized by continuous high-amplitude reflections (Unit 1; Fig. 3), interpreted as representing distal deltaic and draping pelagic sediments. The unit has a variable thickness distribution, being thickest at the base of the Isola Delta, and amounts up to 4 m in the Central Basin and 1–2 m in Maloja Basin, respectively (Fig. 1). Unit 1 encompasses smaller mass-movement deposits that are not individually mapped. The unit drapes a seismically transparent facies corresponding to the “homogenite” deposit (Subunit 2a; Fig. 3) of an extensive megaturbidite in the Central Basin (Blass et al. 2005). The homogeneous mud deposit represents the final phase of sedimentation of very fine particles from suspension in a calm water body (Kastens and Cita 1981; Mulder et al. 2009; Schnellmann et al. 2006) after the Isola Delta collapse. Subunit 2c is characterized by a chaotic seismic facies with scattered diffraction hyperbolae that represents the large bedload transported mass-movement deposit of the Isola Delta collapse. The base of the mass movement is not imaged by the seismic data in the Central Basin. Subunit 2b and Unit 3 are solely found along the seismic stratigraphy of the Lagrev Basin and will be discussed in Sects. 3 and 4.

A sediment core recovered in the Central Basin by Ohlendorf et al. in 1994 (PSS94-2) was analyzed in detail by Blass et al. (2005). The lower part of the core contains an over 6 m thick mass-movement deposit with a base not reached by coring. Below a few cm thick clayey top, the deposit is characterized by a homogenous silty clay in the upper 3.5 m and a very heterogeneous, multiply graded sequence of coarse to very fine sand with varying organic content, and increased variability of magnetic susceptibility (30–320 10^–5^ SI) in the lower 2.5 m (Blass et al. 2005). This large mass-movement deposit was radiocarbon dated to 548–797 cal CE, and interpreted as having originated from a partial collapse of the northern part of the Isola Delta (Blass et al. 2005). Based on high-resolution seismic data and recovered sediment cores, the minimum mass-movement volume was estimated to 6.5 × 10^6^ m^3^ in the Central Basin of Lake Sils (Blass et al. 2005).

## Methods

### Sediment coring

**Fig. 4:**
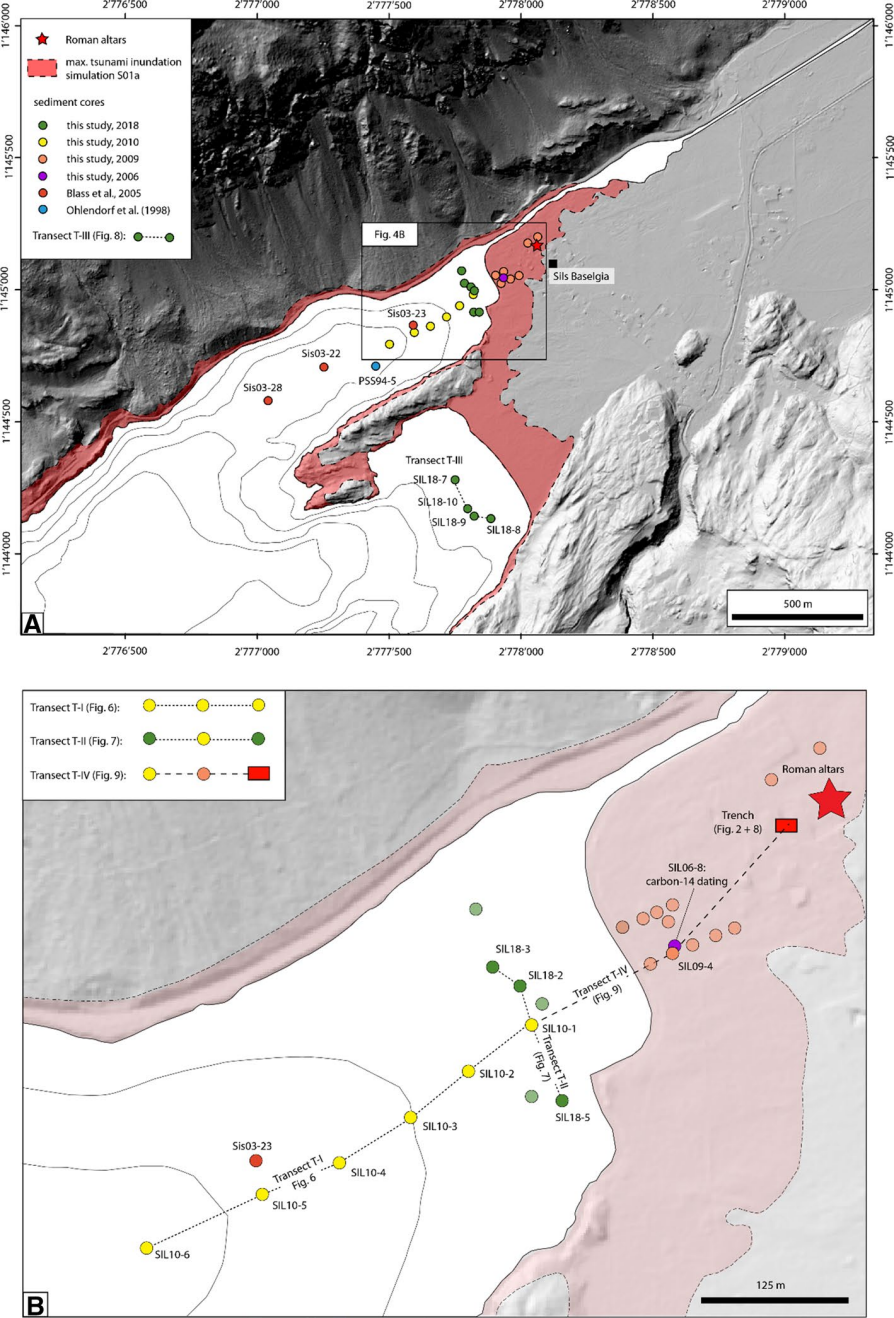
**a** Hillshade map of the northeastern area of Lake Sils (swissALTI3D; swisstopo) shows the excavation site of the four Roman altars in 1964 (red star), sediment core locations (blue: Core PSS94-5 (Ohlendorf 1998); red: Cores Sis03-22; -23; -28 (Blass et al. 2005); orange: terrestrial cores recovered in 2009 with a Geoprobe; yellow: lacustrine sediment cores recovered in 2010; green: shallow-water cores recovered in 2018) and maximum tsunami inundation limit based on numerical tsunami modeling simulation S01a (red shaded area). Transect T-III is shown in Fig. 7. **b** Close-up view of coring location along the Lagrev Basin and on the coastal plain at Lake Sils. Detail view of the different coring Transects T-I, T-II, and T-IV (dashed lines) is illustrated in Figs. 5, 6, and 8. Terrestrial sediment cores were recovered along two transects oriented perpendicular to the lakeshore. Two sediment cores are located more distally close to the excavation site of the Roman altars and the archeological trench made in 2002 (red rectangle; Rageth 2002). Single Core SIL06-8 (violet) was recovered in 2006 and used for radiocarbon dating

A total of 29 sediment cores (13 terrestrial and 16 lake cores) were recovered during multiple field campaigns between 2006 and 2018 (Fig. 4). In 2006, a single, 85 cm long sediment core, SIL06-8 (Fig. 4b), was recovered using a gouge auger at the coastal plain in Sils Baselgia, 50 m from today’s lakeshore. This core bore a 4 cm thick coarse silt deposit at 77 cm depth, overlying an organic-rich peat layer with a sharp basal contact. Radiocarbon dating of the peat layer revealed ages in the period of the delta collapse, motivating us to take 10 additional sediment cores, between 1.8 and 6 m length, which were recovered with a Geoprobe hydraulic-coring system along two onshore transects in 2009. During the same campaign, two further sediment cores were recovered close to the site where the Roman altars were excavated in the year 1964 (Erb et al. 1966). One year later, in 2010, lacustrine sediment cores were collected with a manual percussion-coring system from the frozen lake surface along an orthogonal transect (Transect T-I: Fig. 4b) with 100–500 m distance from the coastal plain at Sils Baselgia. These sediment cores reach core lengths between 0.8 and 2 m and were recovered in water depths from 1.7 to 40 m. Lastly, 10 short sediment cores (0.5–1 m long) were recovered in 2018 along a shoreline-parallel transect in the Lagrev (Transect T-II: 6 sediment cores) and Sils Basin (Transect T-III: 4 sediment cores), respectively. The sediment cores were recovered in a water depth of ~ 2 m at 30–200 m distance from today’s lakeshore with a manual percussion-coring system (Fig. 4). Core recovery of water-saturated sediments was high, and compaction is low when using a percussion-coring system. However, notable compaction was observed with the Geoprobe coring device in very organic-rich sediments that contains fibrous plant fragments. Moreover, low core recovery rates (60–70%) were observed in sandy lithologies with the same coring device.

### Core scanning

All cores were analyzed using a Geotek MSCL-S (Standard Multi Sensor Core Logger), except the gouge auger Core SIL06-8 and Core SIL18-2 which was opened prior to core scanning. Bulk density gamma-ray attenuation (using a 5 mm gamma beam) and magnetic susceptibility were measured with a resolution of 0.5 cm. Sediment cores recovered in 2018 were additionally scanned with a Siemens Somatom Definition AS X-ray computed tomography (CT) scanner at the Institute of Anatomy, University of Bern, prior to opening in order to obtain three-dimensional data of density variations at a voxel size of 100 µm. Computed tomography data were analyzed using the RadiAnt DICOM Viewer software (version 4.6.9.18463). Cores were subsequently split in two halves and line-scan images were captured with the Geotek MSCL-S camera.

### Core analysis: sedimentology, mineralogy, and geochemistry

Macroscopic sediment description of lithologies including color, texture, grain-size distribution, and sediment composition were conducted on split sediment cores and in smear slides. Semiquantitative grain-size distribution was estimated visually on the sediment core for the coarse sediment fraction (sand and gravel) and with a microscope on smear slides for the fine sediment fraction (clay to silt). Further, semiquantitative descriptions of the diatom species assemblages, and mineralogical composition were estimated from smear-slide analysis. Discrete samples were collected from sediment cores to characterize the mineralogical composition by X-ray diffraction (XRD). For this purpose, subsampled sediment samples were dried with a freeze drier and milled. XRD measurements were performed with a powder X-ray Diffractometer (Bruker, AXS D8 Advance) at ETH Zurich. Elemental concentrations of total carbon (TC), total nitrogen (TN), and total sulfur (TS) were measured by gas chromatography (HEKAtech, Euro EA—CHNSO Elemental Analyzer) from discrete sediment subsamples at Eawag in Dübendorf. Total inorganic carbon (TIC) was measured on a coulometer (UIC, CM-5011 CO2 coulometer) at ETH Zurich. Total organic carbon (TOC) was calculated from the difference between measured TC and TIC. Sedimentary C:N ratio was calculated from the ratio of molar TOC and TN concentrations.

### Accelerator mass spectrometry (AMS) ^14^C dating

Terrestrial organic macro-remains from a peat layer were used to date an organic-rich unit below the event deposit in Core SIL10-1 and SIL06-8 with the radiocarbon dating method. Another organic macro-remain was taken from siliciclastic lacustrine sediments above the event deposit in Core SIL10-1. For this purpose, sediment subsamples were wet sieved with deionized water prior to handpicking of terrestrial organic remnants under a binocular loupe. The samples were stored in the freezer until sample preparation for the AMS radiocarbon dating was done. Finally, prepared samples were measured with the Mini RadioCarbon Dating System (MICADAS) at ETH Zurich. Obtained results were calibrated using the OxCal software (version 4.4; Ramsey 2009) and the IntCal20 Northern Hemisphere calibration curve (Reimer et al. 2020).

### Provenance study: mineralogical signature of the major tributaries

Bedload samples were collected from Lake Sils’ major incoming tributaries to characterize the mineralogical signature of the geologically highly complex catchment for provenance analysis of the detrital sediment components observed and analyzed in retrieved sediment cores (Fig. 1). Collected samples were sieved at 2 mm and 63 μm, respectively, to separate the sand-sized sediment fraction. Subsequently, sediment samples were freeze-dried, milled, and homogenized before analysis by powder XRD (X-ray Diffractometer Bruker, AXS D8 Advance) at ETH Zurich.

### Numerical tsunami modeling

Numerical tsunami modeling was performed to assess the potential of the Isola Delta collapse to generate tsunami waves. The modeling approach, which is described in detail in Hilbe and Anselmetti (2015), uses the MassMov2D numerical model (version 0.91; Beguería et al. 2009) for subaquatic mass-movement simulation and the software package GeoClaw (version 4.6.3; Berger et al. 2011) for wave modeling. The input data include a comprehensive topography dataset that was created from the high-resolution swissALTI3D digital elevation model (swisstopo) and a raster dataset interpolated from the isobaths of the 1:25′000 national map (swisstopo). Both were resampled and combined into a single raster dataset with a grid cell size of 5 m. Two different subaqueous mass-movement scenarios with different failed volumes were simulated. The failed volumes, which were estimated from the mass-movement deposit in the lake basin and the present post-failure lake morphology, was added to the present-day Isola Delta. The subaqueous mass movement is simulated as a Bingham plastic in MassMov2D, with rheological parameters taken from Hilbe and Anselmetti (2015). The result of the landslide simulation is fed into GeoClaw as time-dependent changes of the lakebed topography. Tsunami generation, propagation, and inundation are simulated in GeoClaw. Changes of the lakebed are directly transformed to the overlying water column and the water surface using a finite volume method to solve the nonlinear shallow-water equations (George and LeVeque 2006).

### Seismic reflection data

High-resolution, single-channel seismic reflection data were acquired using a 3.5 kHz pinger system with a vertical resolution of ~ 10 cm in the different sub-basins (Maloja Basin (this study); Central, Lagrev, and Sils Basin (Blass et al. 2005)). The acquired seismic reflection data were re-evaluated and interpreted using the seismic interpretation software SMT Kingdom suite 2015. A constant velocity of 1500 m s^−1^ was applied for time-to-depth conversion of both water column and sediment stratigraphy. A special focus was on the characterization of the seismic facies of the mass-movement deposit and its spatial extent in the individual lake basins, as well as on the seismic facies description along the Lagrev Basin.

## Results

### Sedimentology of sediment cores

**Fig. 5:**
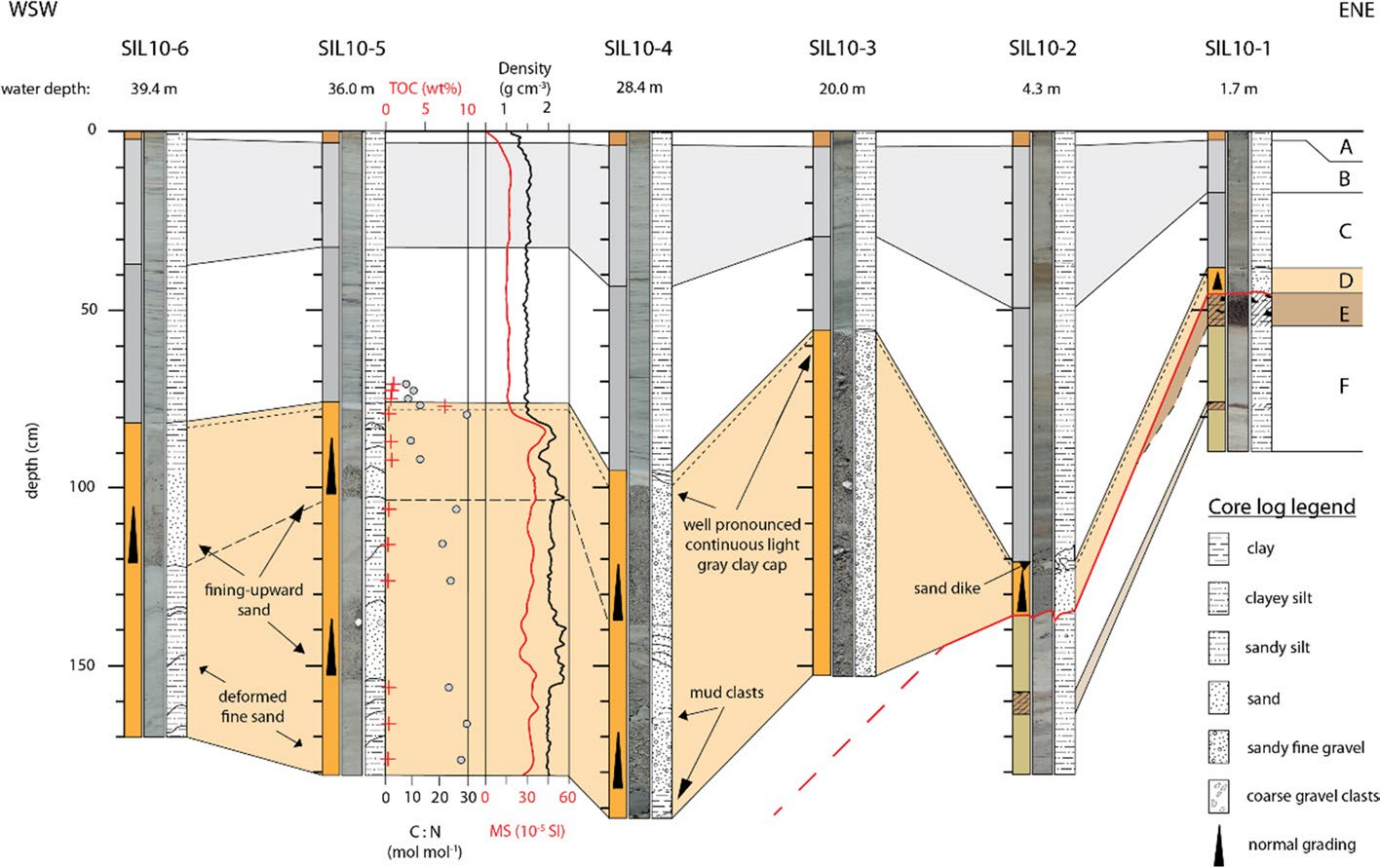
Transect T-I located perpendicular to the shoreline at Sils Baselgia in the Lagrev Basin. Water depth and core ID are indicated above the corresponding sediment cores (see Fig. 4b for core location). The lithostratigraphic succession consists of six sedimentary units (A–F) with lateral thickness variations. Unit D (orange) can be traced along the transect and consists of a heterogeneous sandy deposit with gravels (SIL10-3), a single (SIL10-1 and -2) and multiple (SIL10-4, -5 and -6) fining-upward sequences, and a well-pronounced clay cap. Erosional contact to the underlying lithostratigraphic Units E and F is indicated in Cores SIL10-1 and SIL10-2 (red line). In Cores SIL10-3 to SIL10-6 the underlying sedimentary unit is not reached with coring

**Fig. 6:**
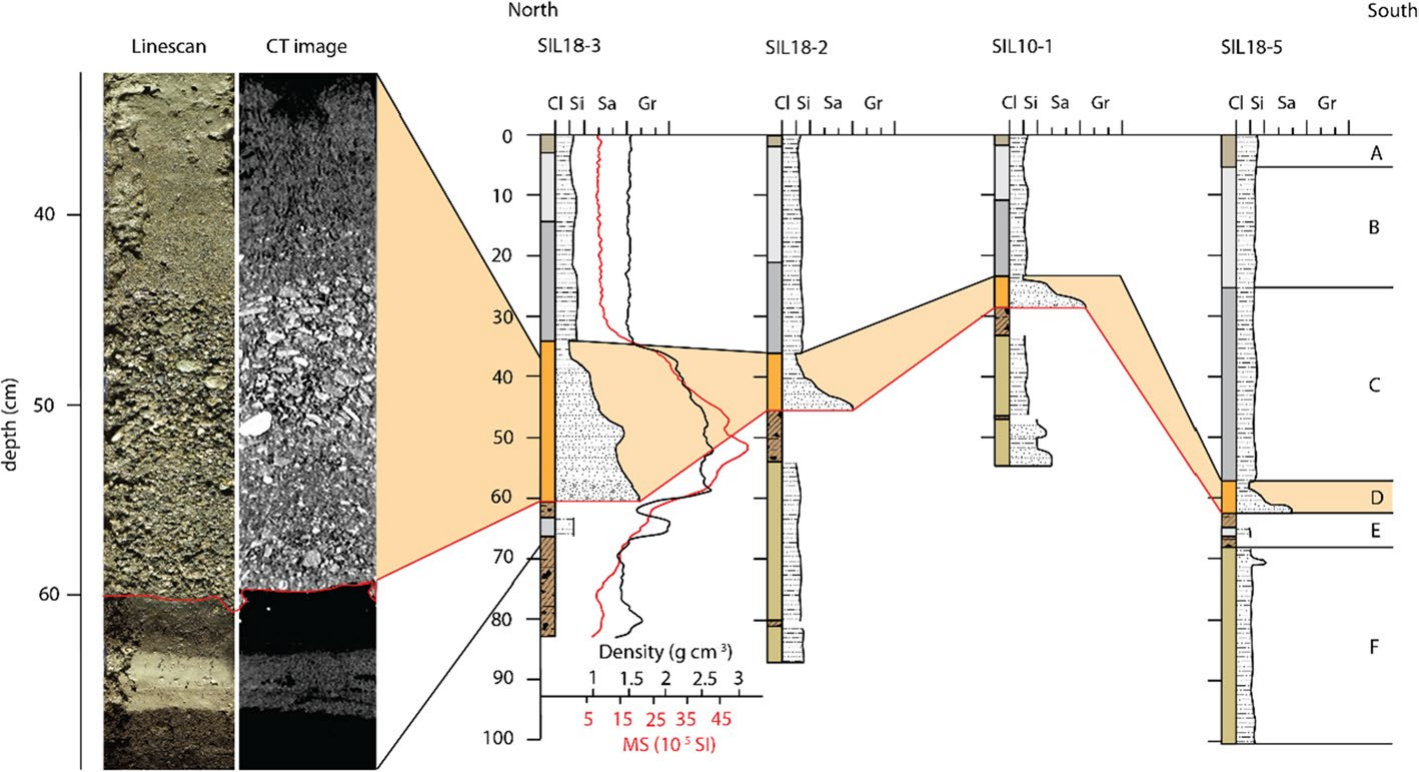
Transect T-II located shore parallel in the Lagrev Basin (see Fig. 4b for core location). Left: Line-scan image and CT-scan image of Core SIL18-3 with a gravelly fining-upward sand deposit (Unit D) overlying with an erosional contact (red line) Unit E. Computed tomography image of Core SIL18-3 shows horizontally bedded gravel clasts in a fining-upward sandy matrix in Unit D. Right: Lateral continuation of the event deposit along the shore of the Lagrev Basin. Bulk density and magnetic susceptibility are indicated for Core SIL18-3

**Fig. 7:**
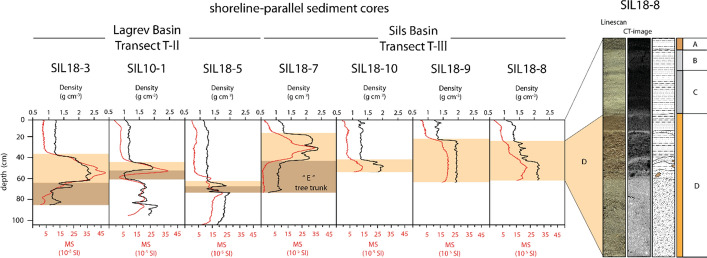
Variation of bulk density and magnetic susceptibility in sediment cores of Transects T-II and T-III. Sediment cores recovered in the Sils Basin (Transect T-III) do not penetrate Unit E, except Core SIL18-7, where a tree trunk was partially cored below (Unit “E”). Core photograph and CT-scan image of Core SIL18-8 show a high-density massive sand in the lower part of Unit D. In the upper part, Unit D is brownish gray with higher concentrations of terrestrial and aquatic macro-remains and two laminae of detrital fine sand and rip-up clasts (brown) are observable

**Fig. 8:**
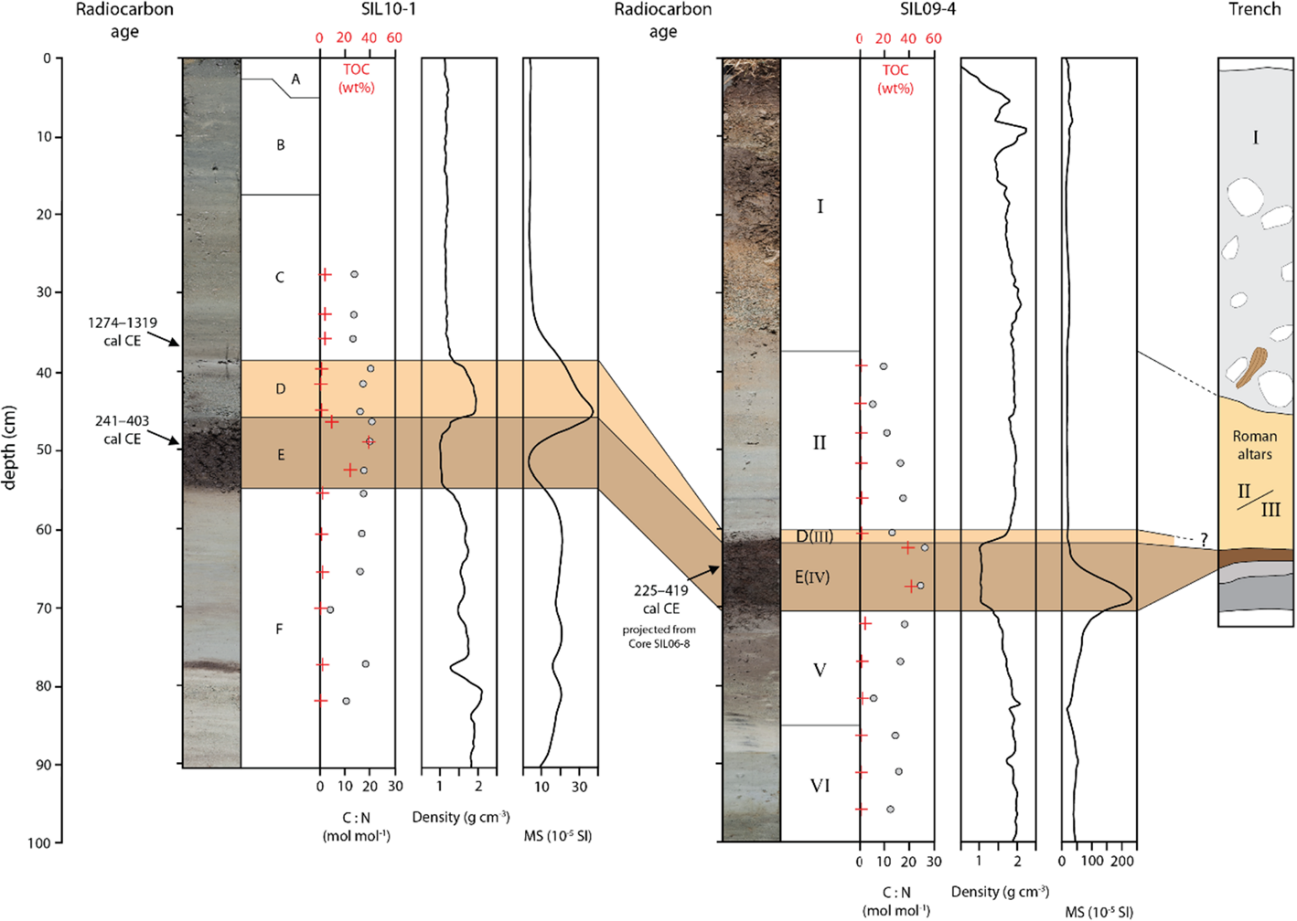
Transect T-IV shows the two Cores SIL10-1 (shallow-water), SIL09-4 (terrestrial), and the archeological trench described in Rageth (2002) (see Fig. 2 for details). The organic-rich Units V and D(V) are ^14^C dated to 241–401 cal CE and 225–419 cal CE, respectively. The base of Unit C was dated to 1274–1319 cal CE. Note that the age from the terrestrial record in Core SIL09-4 is projected from Core SIL06-8 (see Fig. 4b for core locations)

The observed sedimentological composition along four different transects (T-I to T-IV) in the off- and onshore realms is presented in Figs. 5, 6, 7, and 8. The three Transects T-I, T-II, and T-IV contain Core SIL10-1 as the central “anchor” core, linking the different depositional environments of the three transects. Lithological units were correlated along the sediment cores where possible. Definition and numbering of lithological units in all transects follow the same scheme as defined in the Core SIL10-1. However, for terrestrial cores recovered along the low-lying plain at Sils Baselgia (part of Transect T-IV) newly introduced terrestrial-dominated lithological units are labeled with Roman Numerals. Sediment-core location (geographic coordinates) as well as results of the mineralogical and elemental analysis are reported in the Supplementary Material (Appendix A; Tables A1–A6).

#### Transect T-I: shore-perpendicular transect along the Lagrev Basin

Sediment cores of Transect T-I (Fig. 5) along the Lagrev Basin consist of six units in the shallow-water (1.7–4.3 m) and four units in water depths between 20.0 and 39.4 m. To enable comparison with the sedimentological description of Blass et al. (2005) the identical Units A to D were used. Sedimentologically, the uppermost lithological Units A to C are homogeneous and consist of light-gray siliciclastic massive to diffusely laminated silt to very fine sand with abundant diatoms.

The youngest unit is 2–10 cm thick, has the highest abundance of diatoms, smaller grain size, and the highest TOC content (2–3 wt%) compared to Units B and C (Blass et al. 2005). Unit B varies in thickness between 30 and 55 cm, is diffusely laminated and hosts two light-brownish graded detrital layers. Diatom abundance is lower than in Unit A and C. Total organic carbon content is less than 1 wt% in Unit B and ~ 1 wt% in Unit C (Blass et al. 2005). The C:N ratio is around 7–8 mol mol^−1^ in Units A, B, and C (Blass et al. 2005). The silt-sized sediment consists mainly of siliciclastic minerals, carbonate minerals are accessory phases. The mineralogical composition in Unit C consists of chlorite (~ 34 vol%), white mica (~ 26 vol%), quartz (~ 11 vol%), amphibole (~ 9 vol%), K-feldspar (~ 6 vol%), plagioclase (~ 5 vol%), serpentine (~ 5 vol%), and minor amounts of clinopyroxene, calcite, and dolomite.

Unit D is a very heterogeneous and generally coarse-grained deposit that strongly varies in thickness and composition along the transect. The base of the unit is not reached in Cores SIL10-3 to SIL10-6, where it is up to 1 m thick. Toward the shoreline, Unit D thins to 10–15 cm in Cores SIL10-2 and SIL10-1. In Core SIL10-4 and SIL10-5, the unit is characterized by multiple fining-upward sequences composed of a coarse sandy matrix with gravels. A single fining-upward sequence overlies fine sandy silt in SIL10-6. These fining-upward sequences, observed in Cores SIL10-6, SIL10-5, and SIL10-4 consist of clast-supported, coarse sand with angular to sub-rounded gravel. In Core SIL10-4, Unit D hosts deformed and undeformed mud clasts of laminated silt. In Core SIL10-3, Unit D is composed of massive coarse sand with gravel clasts. The top of Unit D is marked, in all cores along the entire transect, by a pronounced and well-traceable light-gray clay cap, with an average thickness of 2 cm. Proximal to the shoreline, Unit D overlies the underlying Unit E with a sharp contact. The C:N ratio in Unit D varies between 10 and 40 mol mol^−1^ in Core SIL10-5. In Core SIL10-2, Unit D directly overlies Unit F with a sharp contact.

Along Transect T-I, Unit E is only present in Core SIL10-1. The unit consists of a 10 cm thick dark-colored organic-rich fibrous peat with finely dispersed white mica and is characterized by high TOC content (30–40 wt%), high TN concentration (2.2 wt%), and a C:N ratio of ~ 20 mol mol^−1^.

Siliciclastic dominated Unit F is characterized by a C:N ratio between 10 and 15 mol mol^−1^, low TOC content (~ 2 wt%), and an absence of diatoms. Grain size varies between coarse silt to medium sand. Units E and F will be discussed in more detail in the description of Transects T-II and T-III.

#### Transect T-II: shallow-water transect along the Lagrev Basin

Sediment cores of the shore-parallel Transect T-II (Fig. 6) were recovered from ~ 2 m water depth in 30–200 m distance from the shoreline along the Lagrev Basin (Fig. 4b). The lithostratigraphic succession consists of six different units, which can be correlated to the deeper-water Transect T-I (Fig. 5). The uppermost Unit A consists of silt-sized brownish-gray sediment with a high TOC content. Units B and C are light gray and consist of homogeneous silt to very fine sand. Units B and C have a lower TOC content compared to Unit A, with a TOC content of 3.3 to 4.5 wt% and a C:N ratio of 13 mol mol^−1^ in Unit C. The silt-sized sediment consists mainly of siliciclastic minerals with carbonate minerals only as accessories.

Unit D has an erosional contact to the underlying Unit E (red line in Fig. 6). The unit consists of a gravelly to sandy base that is overlain by a fining-upward sequence with a silty clay cap at the top. The sequence varies in thickness from 5 to 25 cm along the shore-parallel transect. In Core SIL18-3, the base of Unit D consists of fine gravelly sand, fining-upward to a medium-coarse sand (Fig. 6). Computed tomography scan images highlight horizontally bedded gravel clasts in a coarser section of the generally fining-upward sand at around 44–55 cm depth (Fig. 6). The uppermost part of Unit D is considerably finer and finishes with a pronounced light-gray clay cap. The most dominant mineral phases in Unit D are white mica (~ 30–40 vol%), chlorite (~ 18–36 vol%), quartz (~ 13–30 vol%), plagioclase (~ 6–17 vol%), and K-feldspar (< 1–3 vol%).

The underlying Unit E is a dark brown and very organic-rich (TOC content: ~ 30 wt%) peat deposit containing abundant organic fibrous fragments. The unit has a C:N ratio ranging from 15 to 20 mol mol^−1^. Siliciclastic minerals, mostly white mica, are finely dispersed within the peat horizon. In Cores SIL18-3 and SIL18-5, a 2 to 4 cm thick, greenish-gray silty clay layer occurs within the organic-rich deposit.

Unit F, below the peat, consists of fine to medium siliciclastic sand with a C:N ratio that increases from 10 to 15 mol mol^−1^ upcore. Unlike Units A to C, diatoms are absent in Unit F. The sand-sized detrital components in Unit F consist of mica (~ 33 vol%), chlorite (~ 33 vol%), quartz (~ 17 vol%), and plagioclase (~ 5 vol%). Minor mineral phases comprise amphibole (~ 3 vol%), serpentine (~ 3 vol%), K-feldspar (~ 2 vol%), clinopyroxene (2 vol%), and dolomite (1 vol%).

#### Transect T-III: shallow-water transect along the Sils Basin

A similar lithostratigraphic succession as discussed above occurs along a shore-parallel sediment core transect (Transect T-III: Fig. 7) in the Sils Basin (Fig. 4a). The uppermost strata in the cores are composed of fine-grained siliciclastic silt-sized deposits with abundant diatoms very similar to Units A to C in the Lagrev Basin (Transects T-I and T-II). The thickness of the uppermost Unit A varies between 3 and 8 cm along the transect. Unit A is dark brownish-gray, rich in diatoms, and consists of finer sediment particles compared to Units B and C below, apart from a few distinct coarse high-density laminae. Unit A is characterized by a higher TOC content compared to Units B and C. Units B and C vary markedly in thickness between 10 and 40 cm, are greenish-gray and massive to very diffusely laminated.

A sharp density contrast is observable at the contact to Unit D (Fig. 7). Units A to C have a density of 1.3–1.4 g cm^−3^. In Unit D a very uniform density distribution occurs in Core SIL18-9 (~ 2 g cm^−3^), whereas a more variable density distribution is observed in Cores SIL18-7, -10, and -8 (1.5–2.5 g cm^−3^). The coarse-grained detrital and fining-upward high-density deposit of Unit D occurs along the entire Transect T-III and seems to correspond to Unit D of Transect T-II in the Lagrev Basin (Fig. 7). Accordingly, density generally decreases upcore in Cores SIL18-7, -10, and -8. In contrast to Transect T-II, in Transect T-III Unit D is characterized by discrete layers with increased density, darker colors and coarse sand. The internal multiple stacked fining-upward sequence varies laterally in thickness and has a light-gray clay cap in Cores SIL18-8 and SIL18-10. Vertically oriented, sand-sized, wavy laminae are observable in Unit D in Core SIL18-9. The base of Unit D is only recovered in SIL18-7, where a tree trunk, which was partly recovered in the sediment core, likely represents the equivalent to Unit E in the Lagrev Basin, marked as Unit “E” (Fig. 7).

#### Transect T-IV: cores across the shoreline at Sils Baselgia

Terrestrial sediment cores recovered in the low-lying plain at Sils Baselgia and an archeological trench close to the excavation site of the Roman altars (Fig. 2) show a similar lithological succession. Core SIL09-4 provides one of the best-preserved and longest records. Therefore, Core SIL09-4 is chosen as terrestrial core reference (Fig. 8). Six main lithological units (I–VI) can be distinguished in the terrestrial cores.

The uppermost Unit I varies between 20 and 40 cm in thickness, is yellowish to reddish oxidized, and characterized by heterogeneous organic-rich soil to more clastic-dominated, poorly sorted gravel, and sand. At the top, Unit I consists of 10–20 cm organic-rich soil, which is underlain by a 5 cm thick gravelly layer and another organic-rich soil with a thickness of 10–15 cm. At the base, Unit I is characterized by a fine to coarse sandy gravel with very low TOC content (~ 0.5 wt%) and a C:N ratio of ~ 10 mol mol^−1^. The sand fraction consists of white mica (~ 25–30 vol%), K-feldspar (~ 25–30 vol%), plagioclase (~ 10–20 vol%), chlorite (~ 10–15 vol%), and quartz (~ 10 vol%). Minor abundances are measured for amphibole (~ 3–4 vol%) and serpentine (> 1 vol%). Talc and clinopyroxene are absent or occur only in minor amounts.

Unit II varies in thickness along the transect with largest values (~ 22 cm) at the most proximal location and thinning landward (1–2 cm). Unit II is light gray, diffusely laminated and consists of silt to fine sand with some diatoms. Unit II has a low TOC content (0.4–0.6 wt%) and a variable C:N ratio between 5 and 17 mol mol^−1^. The mineralogical composition consists of abundant K-feldspar (30–45 vol%), white mica (20–28 vol%), quartz (12–15 vol%), plagioclase (10–13 vol%), and chlorite (~ 8 vol%), and minor abundances of amphibole (~ 2 vol%), clinopyroxene (~ 1.5 vol%), and serpentine (< 1 vol%). The boundary to Unit I above is gradual with a reddish to orange oxidized appearance.

Units III and IV can be correlated to Units D and E in Transects T-I and T-II, respectively, and are therefore called D(III) and E(IV) hereafter. However, the thickness of Unit D(III) is variable along the onshore sediment cores. It is generally up to 4 cm thick at a distance of ~ 40 m from today’s shoreline, and thins to 1 cm further away from the shore (~ 80–90 m), until it disappears or is not distinguishable from overlying Unit II (see Supplementary Material for core photos: Appendix A; Figs. A1 and A2). The unit is characterized by poorly sorted siliciclastic silt to fine sand with fragmented diatoms. The diffusely laminated and fining-upward deposit has a high concentration of TOC (~ 1.2 wt%), low TN concentration (~ 0.1 wt%), and a C:N ratio of ~ 13. The deposit shows a sharp to erosional contact to the underlying organic-rich Unit E(IV) (Fig. 8). The distinct clay cap observed in the offshore sediment cores is absent or only very slightly expressed in the onshore setting. Additionally, the upper boundary is gradual and difficult to trace when no clay cap is present. The internal structure of Unit D(III) is variable showing layering at the mm-scale and normal grading. At the base flame structures are visible. However, such structures might also be artifacts related to the coring process.

Unit E(IV) is a dark brown to black porous 10 cm thick peat deposit with fragmented to well-decomposed organic macro-remains. The peat deposit can be correlated along the transect with substantial thickness variations. Total organic carbon content amounts up to 40 wt% with a C:N ratio of 15–25 mol mol^−1^. The transitional contact to the underlying Unit V is characterized by a sharp color change from brownish-black to brownish-gray, a strong decrease of TOC content, and a C:N ratio with a minimum ratio of 10 mol mol^−1^ at 82 cm depth. The fine- to medium-sand siliciclastic fraction becomes more dominant in Unit V. The mineralogical composition of Unit V is characterized by white mica (~ 23 vol%), K-feldspar (~ 20 vol%), quartz (~ 19 vol%), chlorite (~ 18 vol%), and plagioclase (~ 15 vol%) with minor abundances of amphibole (~ 2.5 vol%), serpentine (~ 1 vol%), and clinopyroxene (< 1 vol%). The lowermost Unit VI is composed of a light gray, massive to faintly laminated silt with low TOC content (< 1 wt%), and a C:N ratio of ~ 15 mol mol^−1^.

Rageth (2002) documents a similar lithostratigraphic succession as described above in an archaeological trench at Sils Baselgia at ~ 125 m distance from Core SIL09-4, close to the excavation site of the Roman altars found in 1964 (Figs. 2, 4b and 8). The upper 40–50 cm of the trench consists of an artificial fill containing wood fragments and variable-sized debris with discrete organic-rich horizons, representing recent and medieval cultural horizons and fills (Rageth 2002), and is equivalent to Unit I in Transect T-IV. Below, Rageth (2002) describes a 50–60 cm thick greenish-gray silt-sized sediment package with sandy to gravelly lenses in which the Roman altars most likely were found. This deposit is probably the equivalent of Unit II in Transect T-IV. Below this, Rageth (2002) describes at 60 cm depth a 5–15 cm thick organic-rich deposit with finely dispersed white mica that contains charcoal, bone fragments, processed serpentinite fragments, and Roman brick fragments. This organic-rich unit is very likely the equivalent of Unit E(IV) in Core SIL09-4. The lowermost sediment unit is described as brownish-gray to greenish sandy silt with minor gravel (Rageth 2002). This unit is most likely the equivalent to the lithological Units V and VI (Fig. 8).

**Fig. 9:**
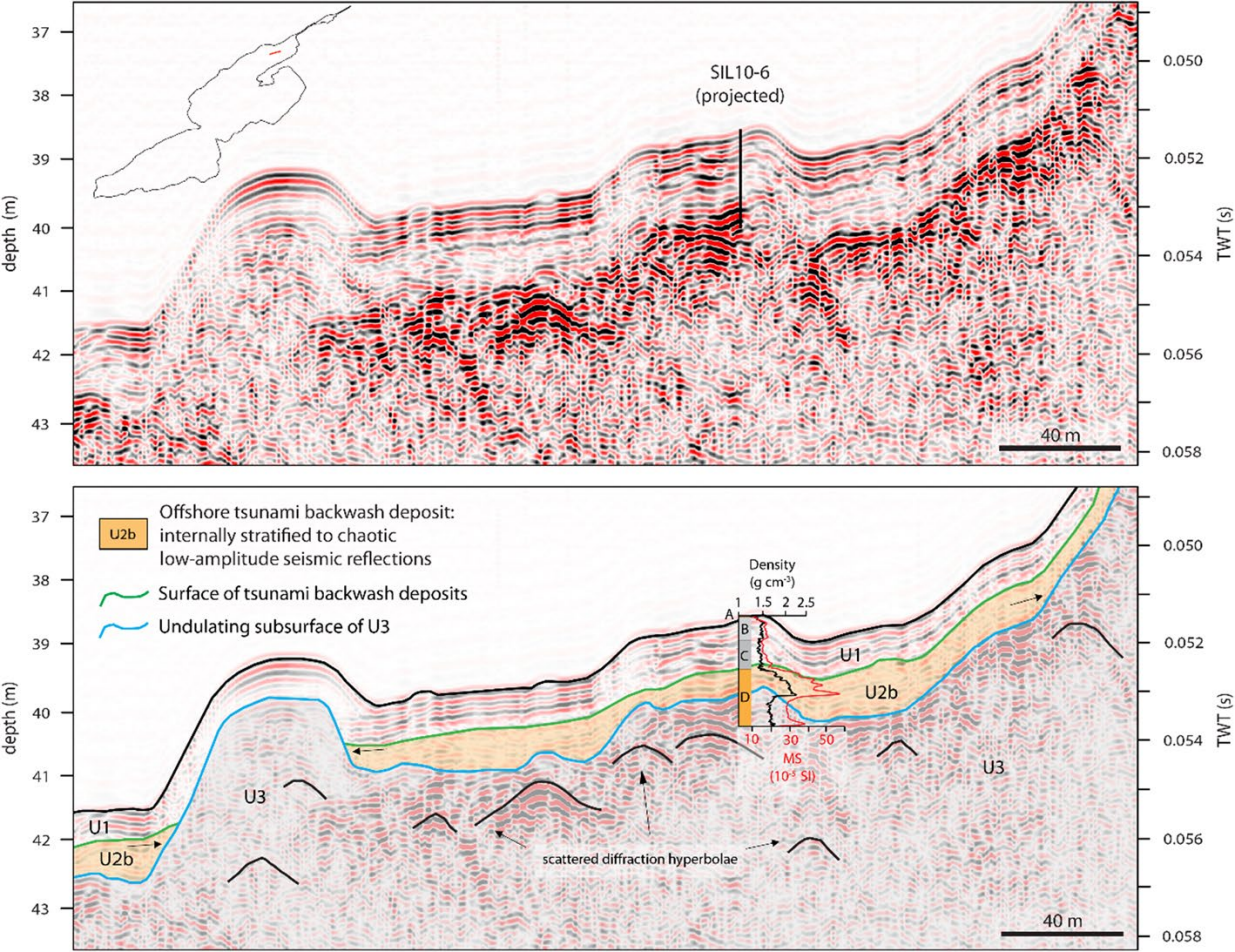
Close-up view of the seismic reflection profile along the Lagrev Basin shown in Fig. 3. Non-interpreted (top) and interpreted (bottom) seismic reflection data and seismic-to-core correlation of Core SIL10-6. The profile location is shown in the inset (top). Overview of the seismic stratigraphy shows continuous high-amplitude reflections of Unit 1 (U1) and chaotic to internally stratified low-amplitude reflections in Unit 2b (U2b). Black arrows mark onlapping of U2b onto surface highs of Unit 3 (U3)

### Seismic reflection data and seismic-to-core correlation

The seismic reflection data of the Central Basin (Fig. 3) shows a 3–4 m thick seismic Unit 1, characterized by continuous high-amplitude parallel reflections representing undisturbed background deposits (Blass et al. 2005). Below seismic Unit 1, an acoustically transparent facies (Subunit 2a, Fig. 3) correlates to the uppermost part of the 548–797 cal CE mass-movement deposit (Blass et al. 2005). No energy is absorbed in the acoustically transparent facies Subunit 2a, most likely indicating fine-grained deposits. Typically, the finest particles of a megaturbidite settle during the final phase of an event, causing a homogenous deposit and a transparent seismic facies (Schnellmann et al. 2002). Below the acoustically transparent seismic facies, a chaotic to patchy facies with some scattered diffraction hyperbolae can be recognized (Subunit 2c, Fig. 3), representing a partly blocky mass-movement unit (e.g., Sammartini et al. 2020). The base of the more than 6 m thick mass-movement deposit is not imaged, probably due to high sand and gas content in the Central Basin. For seismic-to-core correlation, sediment core PSS94-2, recovered in the Central Basin (Fig. 1) by Ohlendorf et al. in 1998, was used. The upper 3.5 m consists of hemipelagic silty clay with intercalations of distal turbidite deposits. The uppermost part of the mass-movement deposit consists of a 4 cm thick light-gray clay cap at the top and a homogeneous silty clay with low variations in density and magnetic susceptibility in the lower 3.5 m that corresponds to the seismic Subunit 2a. The lower seismic Subunit 2c corresponds to a heterogeneous sediment deposit with deformed silty clay sediment packages and several graded sequences with coarse sand to silt (Blass et al. 2005). The base of Unit 2 is not imaged by the seismic data, nor was the base of the mass-movement deposit reached with coring.

Along the Lagrev Basin, a sedimentary body consisting of chaotic to internally stratified low-amplitude seismic reflections (Subunit 2b) is observed above an undulating topography (Unit 3; Fig. 9). The limited continuity reflections of seismic Subunit 2b are parallel to subparallel oriented and show onlapping reflections onto the underlying highs of Unit 3. The sedimentary body can be traced along the entire Lagrev Basin and is characterized by a smooth surface and ponding geometry. Subunit 2b fills geomorphological depressions and reaches thicknesses of 0.5–1 m. The lowermost seismic facies (Unit 3) is characterized by chaotic, low-amplitude seismic reflections with many scattered diffraction hyperbolae and an undulating topography. The base of Unit 3 is not imaged by the seismic data (Fig. 3). Seismic-to-core correlation is based on Core SIL10-6 along the Lagrev Basin (Fig. 9). Seismic Unit 1 corresponds to faintly laminated hemipelagic silt-sized sediment in the upper 80 cm (Units A to C). Below seismic Unit 2b correlates with a sediment package of deformed clayey silt and a fining-upward coarse sand with a light-gray clay cap at the top of lithological Unit D.

### Age of the event deposit

Radiocarbon dating of terrestrial organic macro-remains from the organic-rich peat layer (Unit E) reveals a calibrated radiocarbon age range of 241–403 cal CE in Core SIL10-1 and 225–419 cal CE in Core SIL06-8. The radiocarbon age range is given within a 2σ confidence level, representing a probability of 95.4% (Table 1; Fig. 8). A sample collected in the lowermost part of Unit C is dated to 1274–1319 cal CE. However, due to high δ^13^C (−11.7 ± 1.1 ‰) and very young ^14^C age the sample is rejected. Blass et al. (2005) dated two samples from the uppermost part of the mass-movement deposit in the Central Basin (Core Sis03-23; Poz-5423) and the Maloja Basin (Core Sis03-2; Poz-5424) using the radiocarbon dating method. Calibration of the two ^14^C ages yield a minimum age for the Isola Delta collapse of 548–797 cal CE (Table 1).

**Table 1:** Radiocarbon data and calibrated ages of terrestrial organic macro-remains from Cores SIL10-1 and SIL06-8

Sample code	Core ID	Depth (cm)	Unit	^14^C age ± 1σ (^14^C years BP) ^a^	Calibrated 2σ ranges (cal CE) ^b^	Relative probability (%)	δ^13^C (‰)	Sample material
ETH-40236	SIL10-1	37–38	C	685 ± 30	1274–1319 1359–1389	62.1 33.4	−11.7 ± 1.1	Organic plant remains
ETH-40776	SIL10-1	50–51	E	1745 ± 35	241–403	95.4	−30.5 ± 1.1	Peat: plant remains
ETH-32595	SIL06-8	79	E(IV)	1735 ± 50	225–419	95.4	−25.8 ± 1.2	Peat: 30 plant remains
^c^Poz-5423	Sis03-23	69–72	D (top)	1300 ± 35	654–797	95.4	–	Three small twigs
^c^Poz-5424	Sis03-2	85	D (top)	1465 ± 40	548–652	95.4	–	Leave fragments, small twig

Uncertainties of ^14^C ages refer to 1-sigma uncertainties. Ranges of calibrated ages represent 95.4% probabilities (2σ): ^a^(Stuiver and Polach 1977); ^b^(Ramsey 2009; Reimer et al. 2020); ^c^(Blass et al. 2005)

### Mineralogical composition of the riverine bedload

**Fig. 10:**
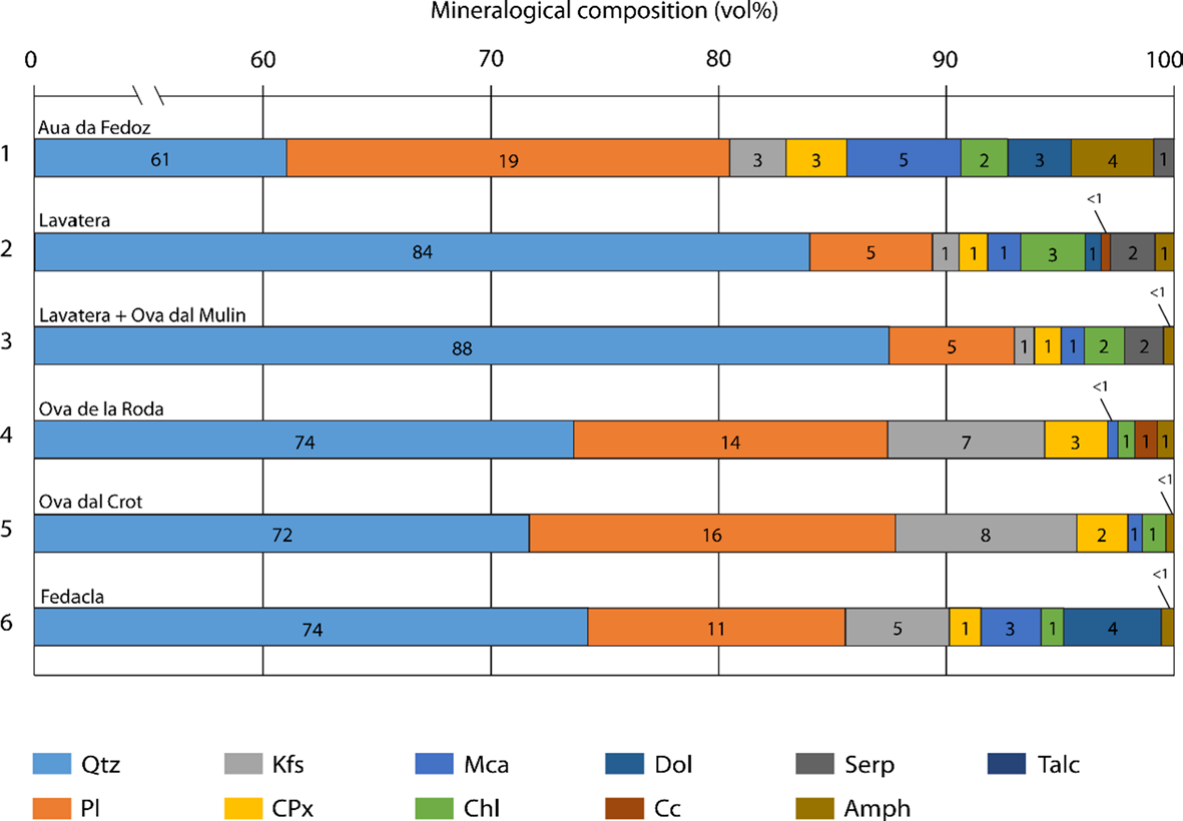
Mineralogical composition of the sand-sized riverbed samples collected at Lake Sils major tributaries given in volume percentage (see Fig. 1 for sample locations). Used mineral abbreviations are *Qtz* quartz, *Pl* plagioclase, *Kfs* K-feldspar, *CPx* clinopyroxene, *Mca* white mica, *Chl* chlorite, *Srp* serpentine, *Am* amphibole, *Tlc* talc, *Dol* dolomite and *Cc* calcite

Mineralogical composition of the sand-sized sediment fraction of Lake Sils tributaries was quantified by X-ray diffraction from the bulk sediment samples taken from the riverbeds of Aua da Fedoz, Lavatera, Ova dal Mulin, Ova de la Roda, Ova dal Crot, and Fedacla (Fig. 1). In general, the mineralogical composition is characterized by a high quartz concentration (60–88 vol%) and minor percentages of plagioclase (5–20 vol%), K-feldspar (< 1–8 vol%), clinopyroxene (1–3 vol%), and mica (< 1–5 vol%). Accessory minerals are chlorite, dolomite, serpentine, and amphibole (Appendix A; Table A3). The Aua de Fedoz, feeding the Isola Delta, is characterized by the lowest concentration of quartz (61 vol%) and highest concentration of mica (5 vol%) and amphibole (3.5 vol%) compared to the northern tributaries and the Fedacla, draining into Lake Silvaplana (Fig. 10). Dolomite is delivered by the southern tributaries Aua de Fedoz (3 vol%) and Fedacla (4.5 vol%), whilst of the northern tributaries it is solely delivered by the Lavatera (< 1 vol%). Serpentine is only delivered by the northern tributaries. Considerable amounts of K-feldspar are delivered by the Ova dal Crot (8 vol%) and Ova de la Roda (7 vol%), draining from the northern slopes.

### Tsunami generation and propagation model

The failed volume of the Isola Delta collapse was estimated on the basis of the deposit volume given by Blass et al. (2005), using geometrical information from bathymetric data, and a tentative reconstruction of the Isola Delta prior to its collapse (see Supplementary Material: Appendix A, Fig.A3). The mass-movement volumes of the two scenarios are considerably smaller than the volume estimated by Blass et al. (2005). This conservative estimate accounts for the fact that volumes estimated based on mapped chaotic seismic facies may be greater than actual failed volumes, because the former may include considerable amounts of deformed pre-existing basin sediments (Hilbe and Anselmetti 2014). Therefore, we modeled the delta failure applying two different mass-movement volumes (Table 2). Due to the lack of detailed information on the pre-failure topography, the estimated volumes were simply added to the present topography, bearing in mind the considerable uncertainties that exist regarding the volume and geometry of the failed mass. Additionally, and for simplicity, only the mass movement toward the Central Basin was simulated, whilst the much smaller mass movement toward the Maloja Basin is neglected. A Bingham plastic rheology was used for the landslide simulation with initial bulk density, yield strength, dynamic viscosity, and constant Manning’s roughness coefficient as given in Table 2.

**Table 2:** Model parameters of the mass-movement, tsunami generation, and propagation simulations

Scenario	Volume (10^6 ^m^3^)	Rheology (constitutive model)	Bulk density (kg m^−3^)	Yield strength (Pa)	Dynamic viscosity (Pa s)	Manning’s roughness coefficient (s m^−1/3^)
S01a	1.71	Bingham plastic	750	5	50	0.03
S01r	1.33	Bingham plastic	750	5	50	0.03

The numerical tsunami model provides an estimation for the order of magnitude of the lacustrine tsunami that might have been generated by the Isola Delta collapse (e.g., inundation, run-up height, depth-averaged velocity, and flow depth). Two different scenarios, S01a and S01r (Table 2), with different initial landslide volumes were run and show comparable results (see Supplementary Material for simulated mass-movement deposit thickness and mass-movement velocity: Appendix A; Figs. A4 and A5). Final mass-movement deposit thickness locally reaches up to 6 m for the two scenarios. Maximum mass-movement thickness locally reaches up to 8.5 m in scenario S01a and 6.5 m in scenario S01r (Fig. A4). Maximum mass-movement velocity of 23 m s^−1^ and 18 m s^−1^ is reached after 14 s in simulation scenario S01a and after 12 s in S01r, respectively, at the Isola Delta slope (Fig. A5).

**Fig. 11:**
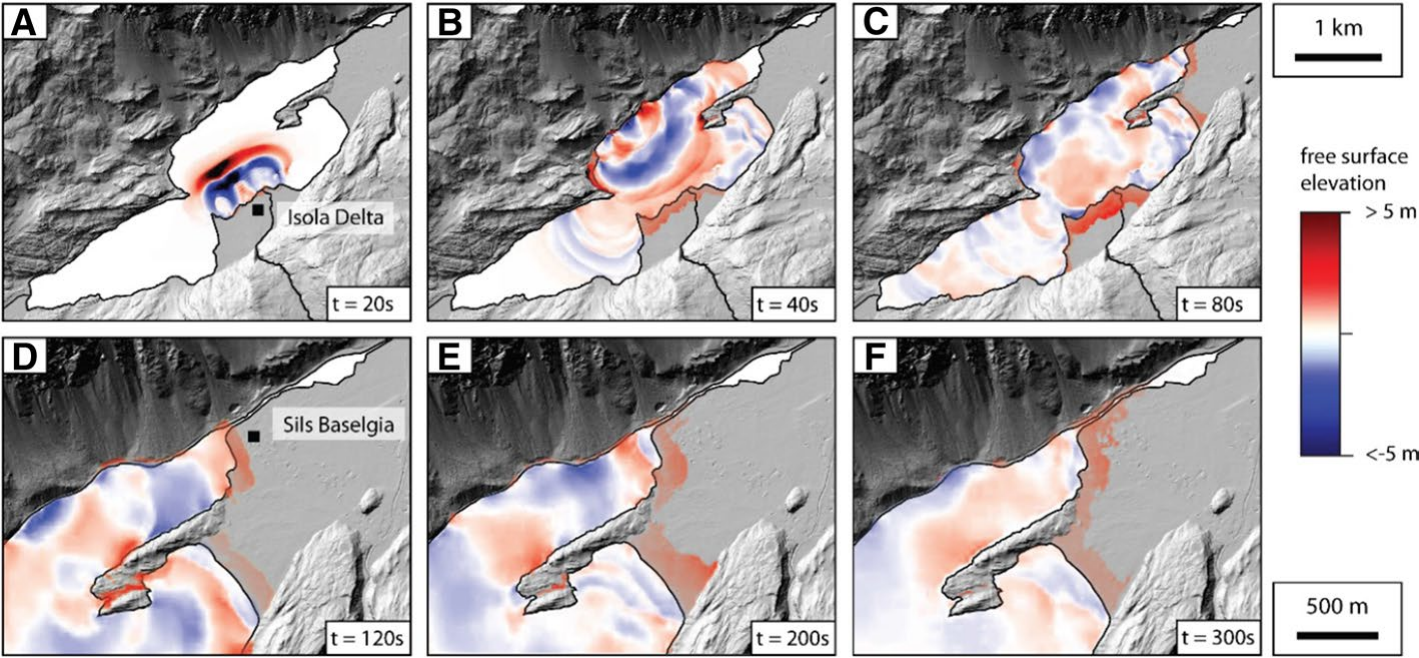
Time steps of the wave amplitude (free surface elevation) of the numerical tsunami propagation model scenario S01a

Tsunami initiation occurs within seconds after the Isola Delta collapse begins (Fig. 11a). A complex interference pattern of waves develops within less than two minutes after slide initiation, spreading along the different sub-basins (Fig. 11b). The wave train reaches the coastal plain at Sils Baselgia after 1 min and inundation reaches up to 200 m from today’s shoreline, with run-up heights of 2–3 m (Fig. 11c).

**Fig. 12:**
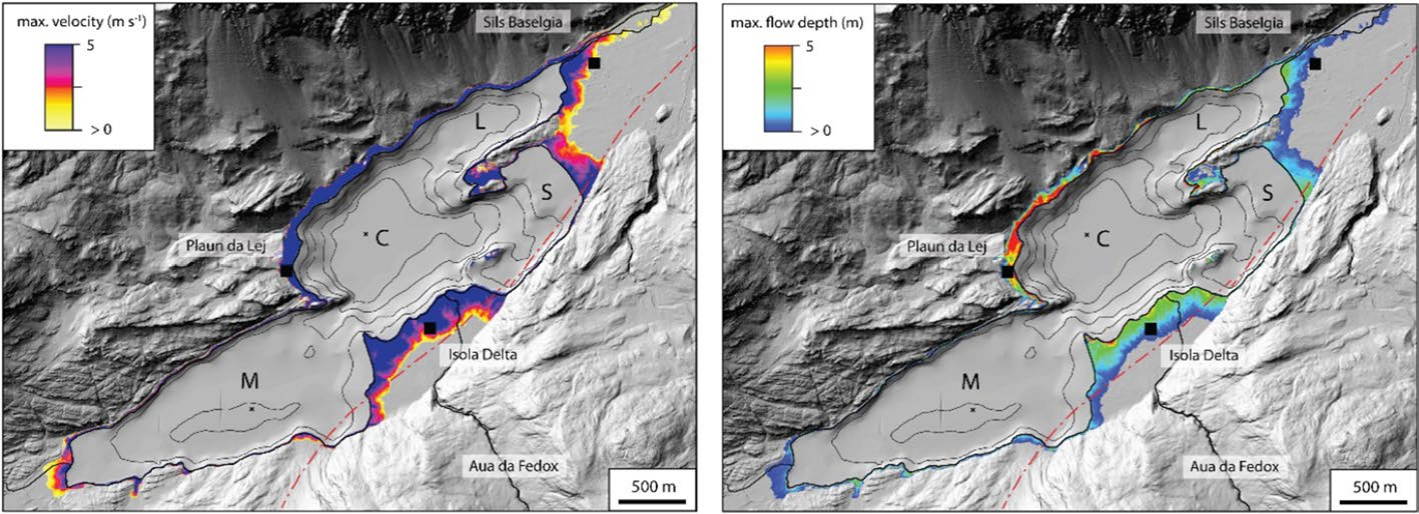
Maximum depth-averaged velocity (left) and maximum flow depth (right) of the numerical tsunami propagation model scenario S01a. Lake Sils’ four sub-basins are indicated with capital letters (*M* Maloja Basin, *C* Central Basin, *L* Lagrev Basin and *S* Sils Basin)

Maximum depth-averaged flow velocity and flow depth reached onshore are shown for scenario S01a in Fig. 12. Highest values are observed on the steep opposite slope at Plaun da Lej along the northwestern shore. Maximum inundation distance (280–300 m) is highest proximal to the tsunami source on the Isola Delta. Here, maximum depth-averaged flow velocity is ~ 5 m s^−1^ and flow depth is ~ 2.5 m. Flow velocity is very high within the first 150 m and drops quickly to near zero at 200 m. Highest maximum flow depths (5 m) are directly observed at the shore, drop quickly to ~ 2.5 m within the first 50 m, and become lower 150 m inland (0–1 m). On the low-lying plain at Sils Baselgia maximum depth-averaged flow velocity drops within the first 100 m from 5 m s^−1^ to 2.5 m s^−1^ (120–170 m) and to near zero in 250 m distance from today’s shoreline. Maximum flow depth is 2.5 m within the first 80 m of inundation and drops continuously to 0 m at the maximum inundation distance at 250–280 m. Maximum depth-averaged velocity (2.5 m s^−1^) and maximum flow depth (< 2.5 m) is generally lower at the low-lying plain adjacent to the Sils Basin. Maximum inundation distance remains around 220 m.

## Discussion

### Sedimentology

A complex, and apparently laterally continuous, event deposit (Units D and D(III)) was identified across different depositional environments within an amphibious transect from onshore areas to the deeper water of Lake Sils. This event layer consists of massive sand and fining-upward gravelly sand sequences with a pronounced clay cap in the shallow-water areas of the Lagrev and Sils Basins. The event deposit transforms into a thicker unit with multiple packages of fining-upward sand to silt and a thicker clay cap toward the deeper water. Large amounts of unconsolidated sediment likely were transported from the northern shore toward the offshore area of the Lagrev Basin by a complex interference of opposing tsunami waves and backwash currents. On the shore-based Transect T-IV, the contact to the underlying peat horizon is marked by an unconformity with a sharp erosional contact (Fig. 8). Horizontally bedded gravel clasts that are embedded in an overall fining-upward sequence indicate high bed shear-stress. Therefore, we assume that vast amounts of unconsolidated sediment were transported as bedload and in suspension from the lake landward. Along the coastal plain, the event layer generally thins and fines landward, with varying thicknesses from several cm in the proximal shore area to 1 cm in the more distal part. Although the coastal plain is relatively flat, with ~ 1 m elevation change along the 100 m long sediment core transect, paleo-microtopography have an important effect on deposit thickness and sedimentary structures of tsunami deposits (Nishimura et al. 2015). Therefore, the variable observed thickness and sedimentological structures are likely a direct consequence of the paleo-microtopography. A pronounced clay cap can only be deposited from standing water over some hours, and therefore, its partial absence can be explained by variations of the paleo-microtopography. The gradual transition toward the upper unit reflects erosion and bioturbation, mechanisms that usually alter tsunami deposits directly after sedimentation (e.g., Goto et al. 2012; Szczuciński 2012; Spiske et al. 2020). The observed trend of decreasing mean grain size landward is consistent with decreasing tsunami velocity with increasing inundation distance, as confirmed by the numerical wave models and was described in various marine tsunami deposits (e.g., Bondevik et al 2005; Dawson 1995; Gelfenbaum and Jaffe 2003). The landward thinning of the event deposit is explained by the decreasing transport capacity of the flow, entraining less sediment the further it travels inland. Moreover, the erosional contacts, the massive and fining-upward sand layers, and horizontally bedded coarse components (Fig. 6) are typical sedimentological signatures found in past and recent coastal plain tsunami deposits. Similar structures have been described for tsunami deposits by Szczuciński et al. (2006), who observed landward thinning of normally graded coarse silt to medium sand along the Andaman Sea coast of Thailand after the 2004 Indian Ocean tsunami. Furthermore, tsunami deposits from the Storegga Slide tsunami are characterized by a remarkably continuous sand layer with evidence of erosion of underlying sediments in Montrose, eastern Scotland (Dawson et al. 1988). For the same event, Bondevik et al. (2003) investigated a generally continuous and normally graded sand layer that thins and fines landward and contains pebbles and organic-rich rip-up clasts. The 1755 Lisbon tsunami deposit at Boca do Rio is characterized as a continuous fining-upward sequence that ranges from coarse sand to clayey sandy silt (Dawson et al. 1995). Although the abovementioned similarities are convincing, tsunami deposits are characterized by a wide range of sedimentological characteristics depending on sediment availability and microtopography (Nishimura et al. 2015). For example, discontinuous sheets of sands are described at Koh Phra Thong, in southern Thailand (Engel and Brückner 2011). Other examples of complex sediment architecture are often caused by shore erosion attributed to tsunami inundation and backwash, as described at many different locations including the Sendai coastal plain after the 2011 Tohoku-oki tsunami (Richmond et al. 2012), at Kalpakkam, India, due to the 2004 Indian Ocean tsunami (Srinivasalu et al. 2007) and at the north coast of Papua New Guinea caused by the 1998 Papua New Guinea Tsunami (Gelfenbaum and Jaffe 2003).

Offshore tsunami deposits in the Sendai Bay following the devastating 2011 Tohoku-Oki tsunami were characterized by distinct layer of beach-derived coarse sand, transported by backwash currents into water depths between 14 and 30 m (e.g., Tamura et al. 2015; Yoshikawa et al. 2015). Channel-like erosion surfaces were identified in offshore seismic profiles in the same area (Yoshikawa et al. 2015) and large subaqueous dunes and bathymetric changes were observed in the Kesennuma Bay, Japan (Haraguchi et al. 2013). Following the 2004 Indian Ocean tsunami multiple studies were conducted on sedimentary deposits on the inner shelf offshore of Khao Lak in the Andaman Sea off the coast of Thailand (e.g., Paris et al. 2010; Sakuna et al. 2012). Sakuna et al. 2012 compared the offshore sedimentary signatures with offshore tsunami deposits in the Portuguese shelf offshore Lisbon (Van den Bergh et al. 2003) and in the Mediterranean (coastal zone of Israel; Goodman-Tchernov et al. 2009 and Augusta Bay, Italy; Smedile et al. 2011). The described sedimentary signatures include a sharp erosional basal contact, thickness between a few cm and m, and grain size in the range of mud to gravel (Sakuna et al. 2012 and references therein). These signatures are well comparable with the offshore deposits in Lake Sils, which are characterized by erosional lower contact, multiple stacks of normal graded coarse sand, and massive gravel.

Based on our sedimentological observations and numerical wave modeling, we hypothesize that the Isola Delta collapse generated a tsunami that impacted the northern lakeshore strongly and transported large amounts of unconsolidated sediment along the lakeshore and toward the deeper basin. The complex interference pattern of waves led to several inundation pulses, so that the back-flowing waters eventually caused downslope currents into the Lagrev Basin. The multiple-pulse flow transported terrestrial sediments into the lake, which were deposited as backwash deposits along the Lagrev Basin. After flow velocities decreased, a distinct clay cap overlying the coarse layer, identified along Transect T-I (Fig. 5), was deposited out of suspension.

Geochemical and mineralogical analysis were conducted to fingerprint the detrital source of the event deposit. Because the geological catchment of Lake Sils is composed of a complex tectonic architecture with many different units, it may be possible to characterize and differentiate detrital components originating from the Aua da Fedoz, the Fedacla, and the northern lakeshore (Fig. 1). The Lavatera and the Ova dal Mulin, draining from the north, are the only riverine bedload samples containing serpentine (1.8–2.0 vol%). The Aua da Fedoz and the Fedacla, draining from the south, deliver dolomite (2.9–4.6 vol%). Comparison between the mineralogical composition of the riverine bedload samples and discrete sediment-core samples indicate that quartz is strongly depleted in the sediment cores (15–30 vol%) relative to the riverine bedload (> 60%). In contrast, mica (20–40 vol%) and chlorite (13–35 vol%) are enriched in the sediment cores, although measured mineral concentrations are very variable compared to the sand-sized riverine bedload sediment fraction. The characterization of the mineralogical composition of the event layer thus supports a local sediment source from the northern slopes. Serpentine is a minor constituent of the detrital event deposit and solely delivered from the northern tributaries of Lake Sils (Fig. 10). Based on the mineralogical composition, we assume that during tsunami propagation large amounts of sediment were remobilized along the northern slopes between Plaun da Lej and Sils Baselgia.

### Age estimation of the deposit

A good age estimation of the depositional timing of the laterally continuous fining-upward and landward-thinning sand event layer is provided by radiocarbon dating on terrestrial organic macro-remains from the peat layer underlying the event deposit. Two radiocarbon samples were retrieved from the organic-rich Unit E in sediment Cores SIL10-1 and SIL09-4 (Fig. 8). Calibration of radiocarbon dating yields ages of 241–403 cal CE and 225–419 cal CE for these core samples, respectively. Assuming that tsunami inundation could substantially erode the alluvial plain close to the shore at Sils Baselgia, and knowing that peat accumulation is a slow process and that the large Isola Delta collapse occurred around 548–797 cal CE (Blass et al. 2005), the obtained radiocarbon ages for the peat layer underlying the event deposit fit well to the proposed mechanism of a tsunamigenic delta collapse, with resulting sediment deposition along the alluvial plain at Sils Baselgia eroding and burying pre-existing soils.

### Tsunami generation and propagation model

Numerical modeling of the delta-slope collapse and associated tsunami waves indicates that a partial Isola Delta collapse would be able to generate a basin-wide tsunami inundating the surrounding nearshore and coastal plain environment. Large amounts of unconsolidated sediment likely were transported from the northern shore toward the offshore area of the Lagrev Basin by a complex interference of opposing tsunami waves and backwash currents. However, model limitations and uncertainties regarding the simulation of tsunami-wave height, run-up and inundation distance need to be considered. For instance, initial delta geometry and landslide volume are not well constrained and were simply added to today’s delta geomorphology in our models. It should also be considered that seismic reflection data and recovered sediment cores in the deep basin do not reach the base of the mass-movement deposit. Therefore, total volume estimation by Blass et al. (2005) is a rough estimate and represents rather a lower bound.

We simulated two conservative delta-collapse scenarios with slightly different initial volumes. Scenarios S01r (1.33 × 10^6^ m^3^) and S01a (1.71 × 10^6^ m^3^) have much lower volumes than the estimated Isola Delta collapse mass-movement deposit in the Central Basin (6.5 × 10^6^ m^3^; Blass et al 2005). Hilbe and Anselmetti (2015) use failed volumes calculated from the scar height and area for modeling the subaqueous mass movement-generated tsunami in Lake Lucerne. These volumes typically amount to half of the volumes observed in the mass-movement deposits. The observed difference is attributed to a suspected incorporation of basin sediments into the mass-movement deposits (Hilbe and Anselmetti 2014). The obtained results, especially in the far field, are satisfactory and comparable with well-documented historical tsunami inundation and run-up (Hilbe and Anselmetti 2015), although the nonlinear shallow-water equations used for the modeling tend to overestimate the height of mass movement-generated tsunamis with shorter wavelengths compared to earthquake-generated tsunamis in the ocean (Lynett 2010). At Lake Sils, neither the total mass-movement deposit in the Central Basin nor the failure scar along the Isola Delta is traceable due to highly dynamic sedimentation mechanism in the deltaic environment.

Subaqueous mass-movement rheology may be another important source of uncertainty. Although the rheological parameters used are equivalent to the parameters used by Hilbe and Anselmetti (2015) for the Muota Delta collapse, which is thought to be comparable to the Isola Delta collapse, evidence revealing the kinematics of submarine landslides remain scarce (Løvholt et al. 2015). Yet, tsunami generation, amplitude, and wavelength are influenced by mass-movement kinematic parameters (Løvholt et al. 2015) and mainly determined by the volume, the initial acceleration, and the maximum velocity (Harbitz et al. 2006). Maximum mass-movement velocities simulated (16–24 m s^−1^) are comparable to other simulated subaqueous mass movement-generated tsunamis in the same order of volume (e.g., 2014 Statland Tsunami, Norway; Glimsdal et al. 2016). Sensitivity analysis of the mass-movement rheology parameters for the Muota Delta collapse-generated tsunami at Lake Lucerne indicates that the initial volume and mass-movement geometry as well as the dynamic viscosity are the most important parameters controlling tsunami run-up and wave height (Hilbe and Anselmetti 2015). Although shore texture and land cover, expressed as bottom roughness, can significantly affect tsunami inundation and velocity (Kaiser et al. 2011), and we do not know the land cover at the time of the collapse, a constant Manning’s value was used for bottom roughness of 0.03 s m^−1/3^ on the alluvial plain.

Tsunami sediment erosion and transport capacity depend mainly on bed shear-stress and shear velocity (e.g., Ontowirjo et al. 2013; Paris et al. 2010). Simulated tsunami flow velocity ranges from 0 to 5 m s^−1^ along the alluvial plain at Sils Baselgia. Such flow velocities exceed critical threshold conditions for incipient motion of silt-, sand- and fine gravel-sized siliciclastic particles. Therefore, erosion of sediment particles along the slope and in the foreshore area, as well as erosion of the paleosol along the alluvial plain may be caused due to tsunami inundation. Silt- and sand-sized particles are transported in suspension, larger blocks and gravel as bedload fraction. With decreasing velocity, a normally graded succession (Units D and D(III)) is deposited from sediment falling out of suspension in the onshore and in the shallow-water setting, respectively, whereas multiple deposits of fining-upward sequences along Transect T-I probably were deposited during pulse-like localized backwash currents.

### Other potential event deposit mechanisms

Besides the tsunamigenic Isola Delta collapse, some other sedimentary processes could potentially have caused the investigated event deposit. In the following, we discuss why we consider these mechanisms less plausible:

(i)* Debris flows:* Although debris flows show a wide range of characteristics, they are commonly characterized by the sediment transport of particles ranging from clay to large boulders in a dense viscous flow. Water velocities range between 0.5 and 20 m s^−1^ (Takahashi 1981) on alluvial or debris cones with a slope usually between 4 and 8° containing channels with well-developed boulder-rich lateral levees (Takahashi 1981). The sedimentary deposits consist of poorly sorted mixture of particles and are generally fine distal from the source. In the lowermost areas off-fan deposition of winnowed fines may be observable (Blair and McPherson 1998).

(ii)* Rockslides:* Rockslides are characterized by transport of debris from disaggregated bedrock with a relatively shallow-seated glide plane (Voight et al. 1981). The rate of movement can be very slow to extremely rapid, with an abrupt disintegration of the slope (Allen 2009). The associated sedimentary deposits are usually very poorly sorted and often contain wooden debris and tree trunks. However, sorting is more effective when large amounts of water are involved, and the rockslide gradually transforms into a debris flow.

(iii)* Lake-level changes:* Submerged paleo-shorelines are formed by gradual water-level rise in a transgressive phase and may leave site-specific depositional signatures in the geological record. Often these deposits consist of a fining-upward sequence above an unconformity with an erosional to sharp contact due to a relatively abrupt water-level rise (e.g., Merzeraud et al. 2019). Such a transgressional facies pattern is characterized by a change from coastal sediments to coarse clastic nearshore deposits, which are overlain by fine-grained deposits.

These geological processes may generate similar depositional signatures as the observed fining-upward and landward thinning and fining Unit D at the coast of Lake Sils. But certain aspects clearly speak against their genesis. (i) A debris-flow cone is characterized by a decreasing particle size distal to its origin. At Lake Sils the Fedacla river, draining the Val Fex, certainly flooded the alluvial cone at Sils repeatedly. Associated flood layers were described by Blass et al. (2005) in recovered sediment cores from Lake Sils. But the investigated Unit D is thinning and fining landward, therefore a deposition from a debris flow can be excluded. Additionally, the southern tributaries have different sediment provenance than the mineralogical composition observed in the event deposit. (ii) Rockslides originate from the northern shore at Lake Sils, as indicated by several talus and block deposits on the slopes of Piz Lagrev. Associated sedimentary deposits are expected to be less sorted due to the proximity of the steep slope. Moreover, these deposits would only occur in the proximal Lagrev Basin as they would not reach the distant Sils Basin (Fig. 1). (iii) A lake-level rise is indicated by the buried peat layer (Unit E), but an abrupt rise is considered not to be able to transport the amount of sediment needed to deposit Unit D in the lacustrine setting. Moreover, none of these alternate processes would generate the observed clay cap at the top of Unit D in Transects T-I, T-II, and T-III, which clearly indicates an event deposition with very large suspension involved, favoring a large delta collapse.

### Unifying hypothesis: delta collapse-generated tsunami

Tsunamigenic delta collapse are historically reported in Lake Geneva (563 CE Rhone Delta collapse (Kremer et al. 2012), Lake Lucerne (1687 Muota Delta collapse (Hilbe and Anselmetti 2014)), and Lake Brienz (1996 Aare Delta collapse (Girardclos et al. 2007). Compared to these tsunamigenic delta collapses, the observed depositional volume of the Isola Delta mass movement is best comparable with the 1687 Muota Delta collapse (14 × 10^6^ m^3^) in Lake Lucerne (Hilbe and Anselmetti 2015). Historical documents of this event report a tsunami run-up of 5 m at the opposing lakeshore, about 1.2 km distant, and severe demolition of coastal infrastructures (Dietrich 1689). The shore behind the delta was considerable damaged in the proximal area of the village Brunnen by two main pulses of inundation and subsequent backwash currents (Dietrich 1689).

Although the (conservatively calculated) mass-movement volume involved in the Isola Delta collapse is almost half of the volume of the Muota Delta collapse at Lake Lucerne, delta morphology and major constituents are thought to be very similar. This is especially important with respect to mass-movement rheology and tsunami generation mechanism. Because tsunami modeling results obtained for the Muota Delta collapse correlate well with historical documented wave parameters (Hilbe and Anselmetti 2015), the simulated Isola Delta collapse-generated tsunami, calculated using the same numerical codes, is thought to provide a realistic frame. Further, previous studies have shown that tsunamis generated by submarine landslides often have very large run-up heights close to the source area and are most dangerous when generated in shallow waters (Harbitz et al. 2006).

Despite the lack of direct historical reports documenting a tsunami event in Lake Sils, the observed sedimentological succession in sediment cores from the onshore and shallow-water setting provides insights into a severe depositional event. Radiocarbon dating of the event underlying peat layer indicates that the event occurred after 225–419 cal CE. The large Isola Delta collapse with a depositional volume of at least 6.5 × 10^6^ m^3^ and dated to 548–797 cal CE (Blass et al. 2005) is the best and obvious candidate to cause the event deposits. Firstly, it postdates the organic-rich sediments underlying the event layer by a short period. Secondly, the observed sedimentological characteristics points toward tsunami sediment transport and deposition. Thirdly, sediment provenance indicates that a local sediment source from the northern shore is very likely the major sediment contributor for the observed event deposit along the Lagrev Basin and the coastal plain. Fourthly, our numerical tsunami simulations indicate that the mass-movement volume of the Isola Delta collapse is sufficiently large to displace large amounts of water so that the required inundation and run-up can be reached.

### Triggering mechanism of the Isola Delta collapse

Subaquatic mass movements may have variable trigger mechanisms, ranging from seismic to climatic causes (Kremer et al. 2017). Multiple synchronous mass movements on statically stable lateral lake slopes are usually triggered by seismic shaking (Kremer et al. 2017), while potentially unstable delta slopes may also collapse spontaneously (Girardclos et al. 2007; Hilbe and Anselmetti 2014). The timing of the Isola Delta collapse (548–797 cal CE) coincides, within the resolution of the radiocarbon dating method, with multiple subaquatic mass movements identified on reflection seismic data and sediment cores in nearby Lake Silvaplana at 571–650 cal CE (Bellwald 2012 in Kremer et al. 2017). Moreover, a large mass-movement deposit was identified in Lake Como 60 km to the south and dated to ~ 530 CE (Fanetti et al. 2008). Such multiple coevally triggered mass movements deposited along the same horizon within a restricted basin and in different lakes are strong evidence for an earthquake-triggered collapse (Kremer et al. 2017; Schnellmann et al. 2002), so that the Lake Sils event may have been also triggered by a strong regional seismic event.

## Conclusions

A large subaqueous delta collapse emplaced a mass-movement deposit with a maximum thickness of more than 6 m and a total estimated minimum volume of 6.5 × 10^6^ m^3^ in the Central Basin of Lake Sils around 548–797 cal CE (Blass et al. 2005). Our sedimentological analysis of sediment cores retrieved on the coastal plain and in the shallow water of Lake Sils supports the hypothesis that this mass movement generated a basin-wide tsunami that was able to substantially erode unconsolidated sediment along the lakeshores, especially along the northern shore. Recovered sediment cores show a prominent fining-upward sequence above an erosional sharp contact to the underlying paleosol that can be correlated across all sediment core transects. Based on radiocarbon dating, this paleosol was formed 225–419 cal CE, indicating a 2–3 m lower lake level at the time. The overlying coarse-grained event deposit consists of horizontally bedded gravel in a fining-upward sandy matrix thinning and fining landward. Cores recovered in the deeper part of the Lagrev Basin host a tsunami backwash deposit that contains multiple successions of fining-upward sequences with underlying erosional contacts. A clay cap marks the top of the event deposit, indicating sedimentation of the finest particles from suspension in the latest phase of the event.

Based on numerical tsunami-wave modeling, the Isola Delta collapse could have mobilized enough sediment to generate a basin-wide tsunami. Our simulations indicate that the delta collapse generated an initial wave with a free surface elevation exceeding 3 m in the direction of the emerging delta-slope failure. The wave train traveled along the main direction in which the slope failed and inundated the opposite shore. Due to restricted basin morphology, complex interference of waves caused them to spread along the different basins, resulting in a main wave direction traveling into the Lagrev and Sils Basins. These waves inundated the alluvial plain at Sils Baselgia with a maximum run-up of 2–3 m up to 200 m inland with initial flow velocities of up to ~ 5 m s^−1^. Although the performed numerical tsunami simulations suggest that the area where the Roman altars have been excavated was flooded, it remains questionable whether the entire fine-grained deposits embedding the altars represent tsunami deposits. Yet, it is likely that the Roman altars were tilted and displaced by the tsunami. They eventually became buried by deposits from the inundation as well as by overlying lake sediments, indicating also a post-event lake-level rise of over 2 m.

Because the described megaturbidite and its associated shallow-water and coastal deposits are the only identified major mass-movement deposit in the Central Basin and around Lake Sils (reflection seismic data and sediment cores do not penetrate further into the postglacial fill of the deep basin), recurrence rates of similar delta failures cannot be constrained. Nevertheless, high sedimentation rates and oversteepening in delta areas such as the Isola Delta are the ideal preconditioning factors for causing multiple failures in relatively short time, as was observed in other lacustrine case studies (e.g., Girardclos et al. 2007). These processes and phenomena need to be taken into consideration when evaluating the hazard potential of future subaqueous delta collapse-generated tsunamis in inhabited areas.

## Supplementary Information

Below is the link to the electronic supplementary material.

Supplementary file1 (DOCX 4377 kb)
