# Supplementary material for: A tsunamigenic delta collapse and its associated tsunami deposits in and around Lake Sils, Switzerland
Source: Nat Hazards (Dordr). 2021 Feb 8;107(2):1069–103. doi: 10.1007/s11069-021-04533-y (PMC8550072; doi:10.1007/s11069-021-04533-y)
Supplement: Supplementary file 1 — Supplementary file1 (DOCX 4377 kb) [file 11069_2021_4533_MOESM1_ESM.docx]

**Appendix A**

**A tsunamigenic delta collapse and its associated deposits in and around Lake Sils, Switzerland**

Valentin Nigg^1*^, Stephan Wohlwend^2^, Michael Hilbe^1^, Benjamin Bellwald^3^, Stefano C. Fabbri^1^, Gregory F. de Souza^4^, Florian Donau^2^, Reto Grischott^2^, Michael Strasser^5^, Flavio S. Anselmetti^1^

^1^ Institute of Geological Sciences and Oeschger Centre for Climate Change Research, University of Bern, Baltzerstrasse 1+3, 3012 Bern, Switzerland

^2^ Geological Institute, ETH Zurich, Sonneggstrasse 5, 8092 Zürich, Switzerland

^3^ Volcanic Basin Petroleum Research (VBPR), Høienhald, Blindernveien 5, 0361 Oslo, Norway

^4^ Institute of Geochemistry and Petrology, ETH Zurich, Clausiusstrasse 25, 8092 Zürich, Switzerland

^5^ Department of Geology, University of Innsbruck, Innrain 52, 6020 Innsbruck, Austria

^*^ Corresponding author: Valentin Nigg, [valentin.nigg@geo.unibe.ch](mailto:valentin.nigg@geo.unibe.ch)

**Onshore sediment cores
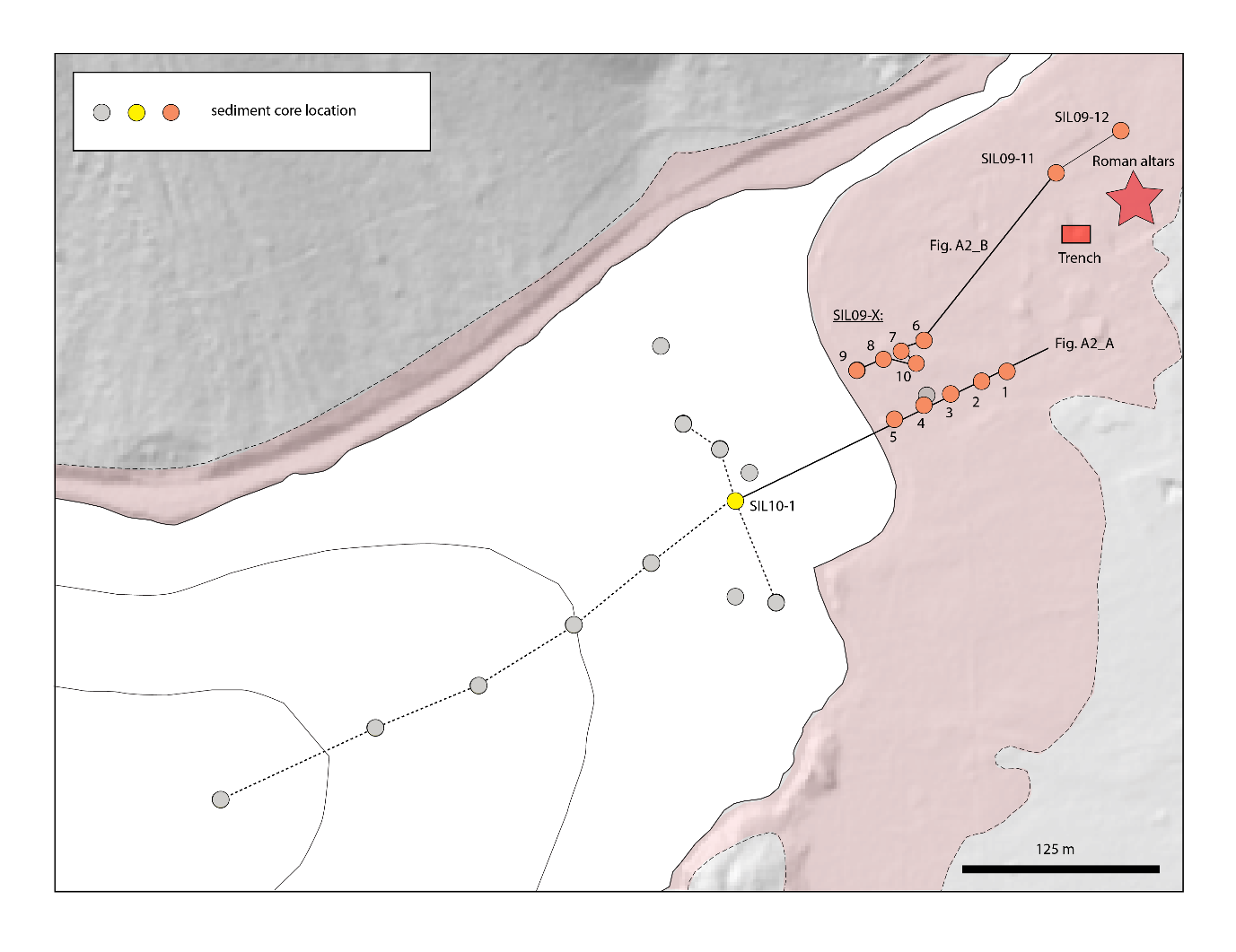
**

**Fig. A1** Detail map of the coastal plain at show onshore sediment core location and core label

**
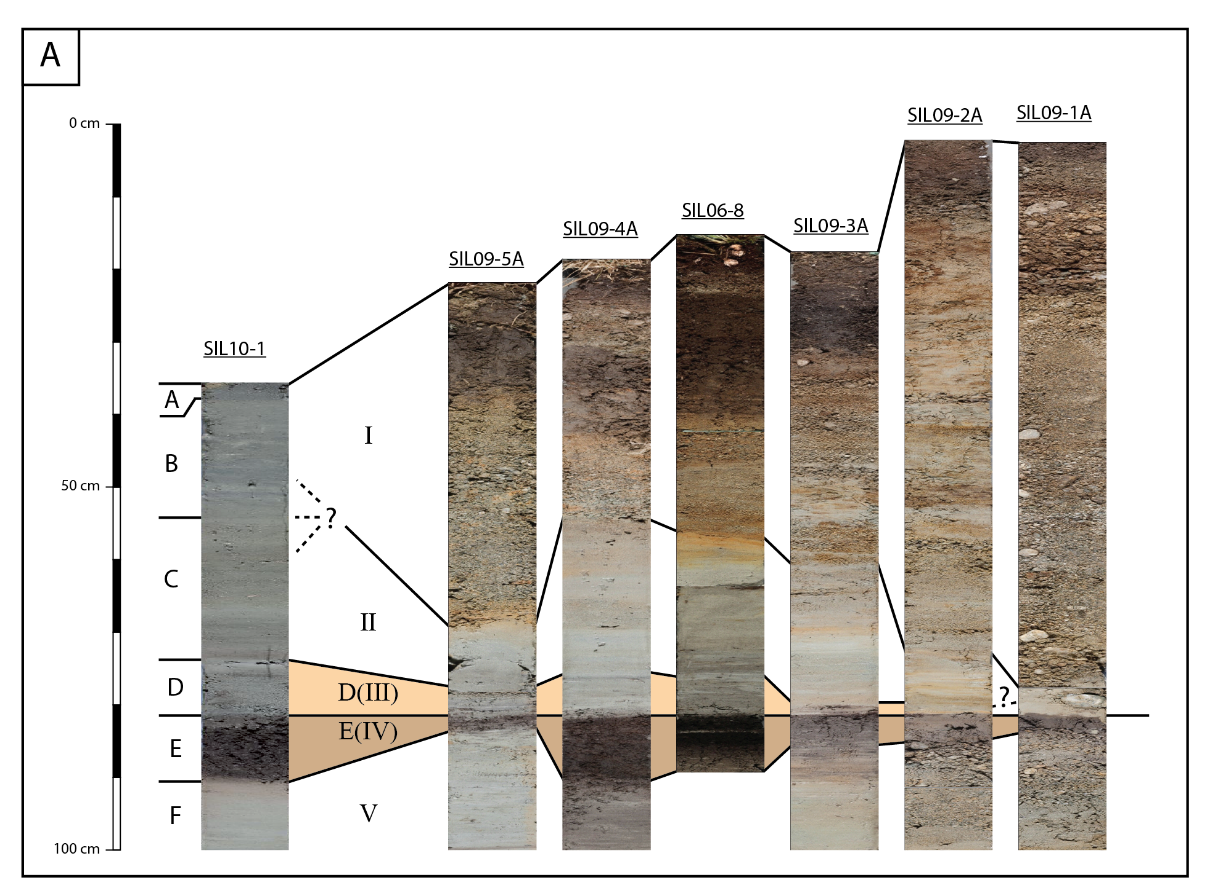

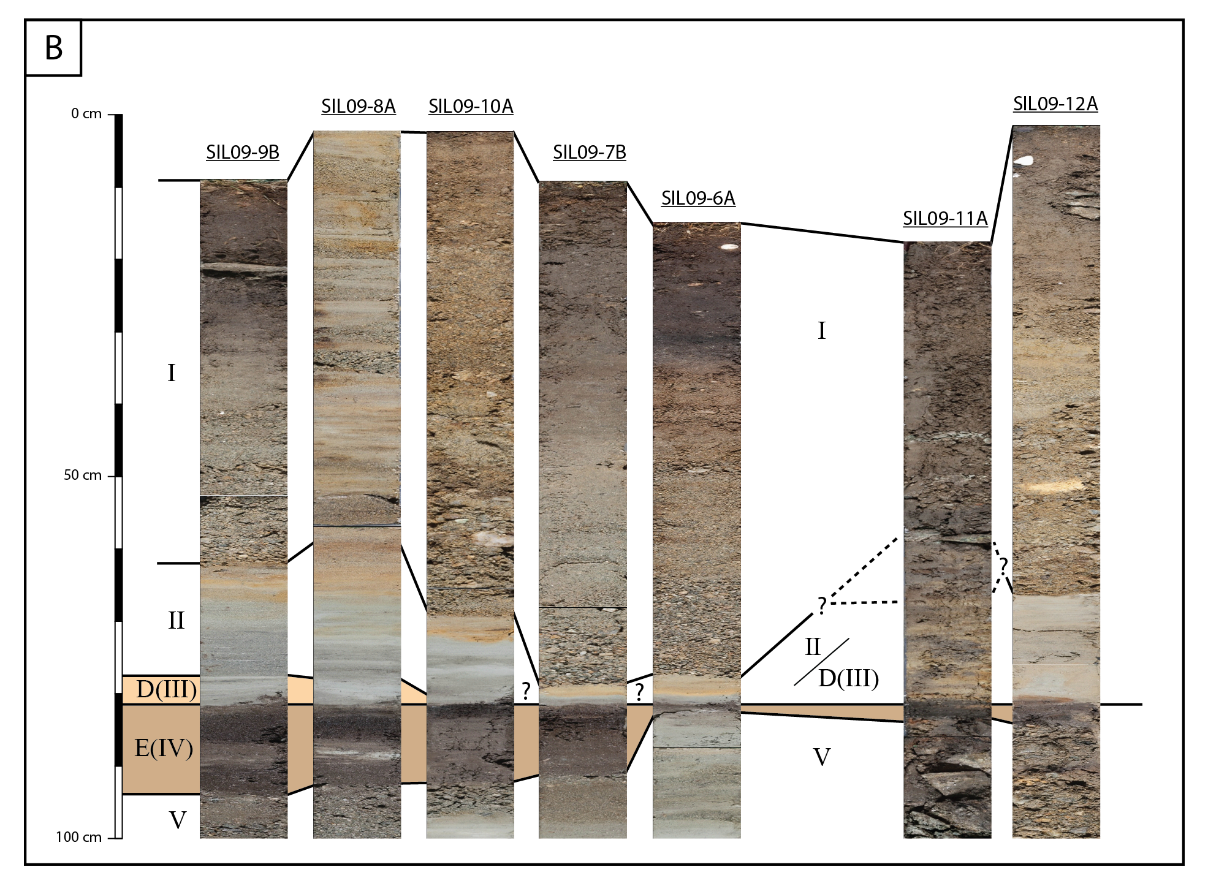
**

**Fig. A2** Core line scan images and lithological interpretation of onshore sediments (see Fig. A1 for core location). Lithological units are labelled according to the descriptions in the manuscript.

**Numerical tsunami modeling – total failed volume estimation**

For the volume estimation of the Isola Delta collapse two different scenarios were calculated (Fig. A1). The total failed volume is 1.33 10^6^ m^3^ (scenario S01r) and 1.71 10^6^ m^3^ (scenario S01a), respectively. The total volume consists of three individual packages with different initial heights above todays lake floor.


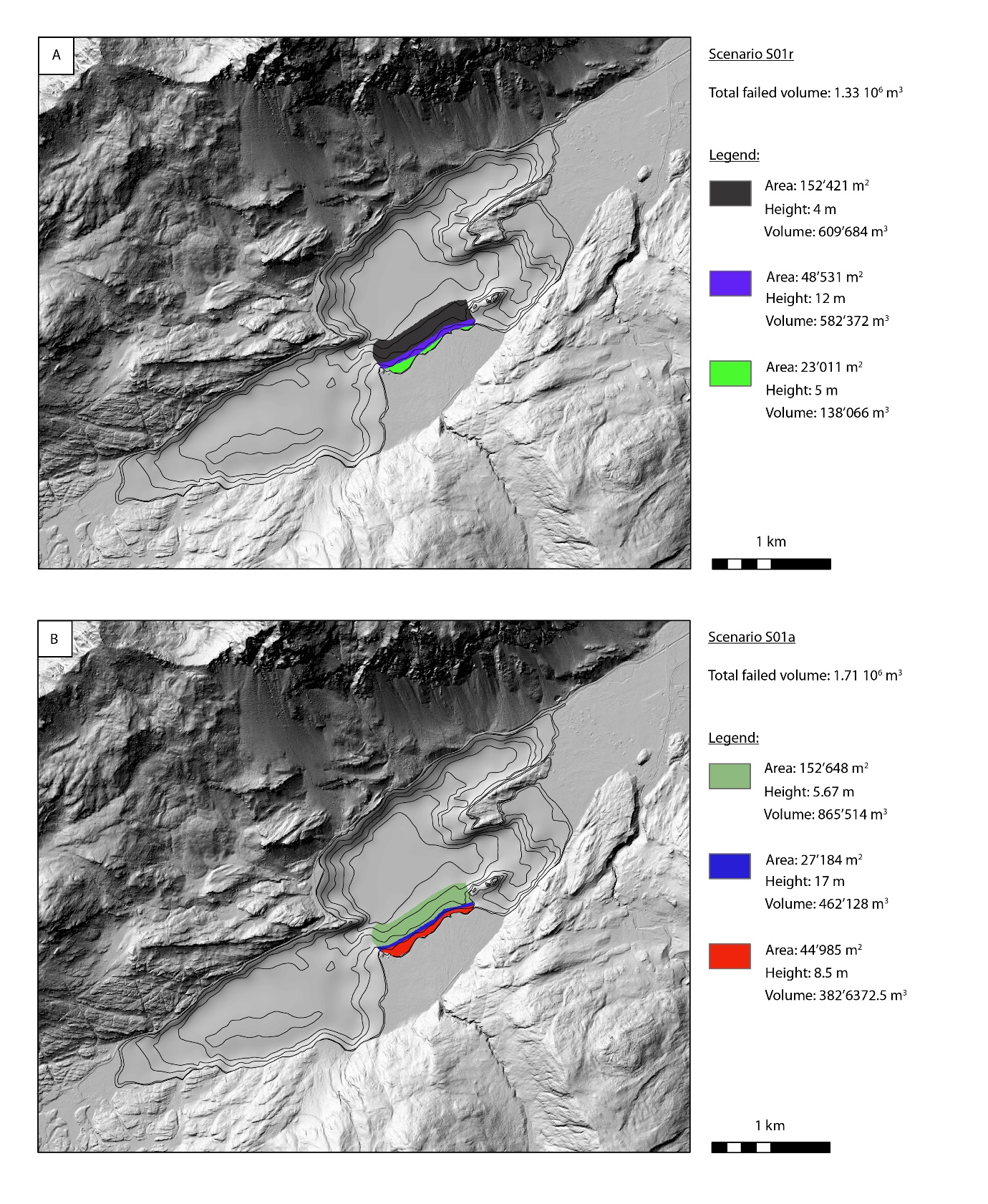


**Fig. A3** Total failed delta volume of the two different numerical modeled scenario S01r (A) and S01a (B)

**
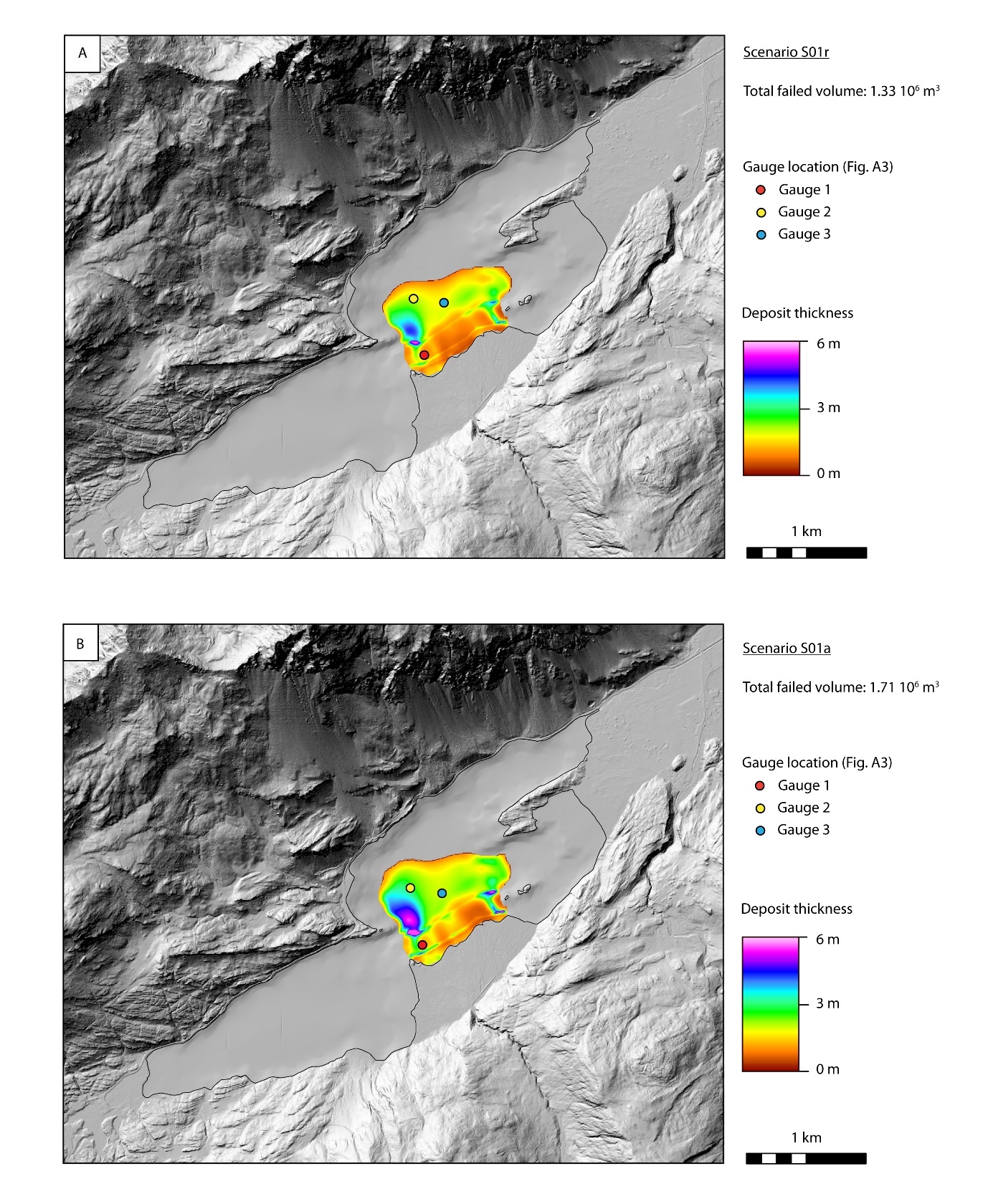
**

**Fig. A4** Simulated mass-movement deposit of the two different numerical simulations S01r and S01a, with the initial volumes shown in Fig. A1

**
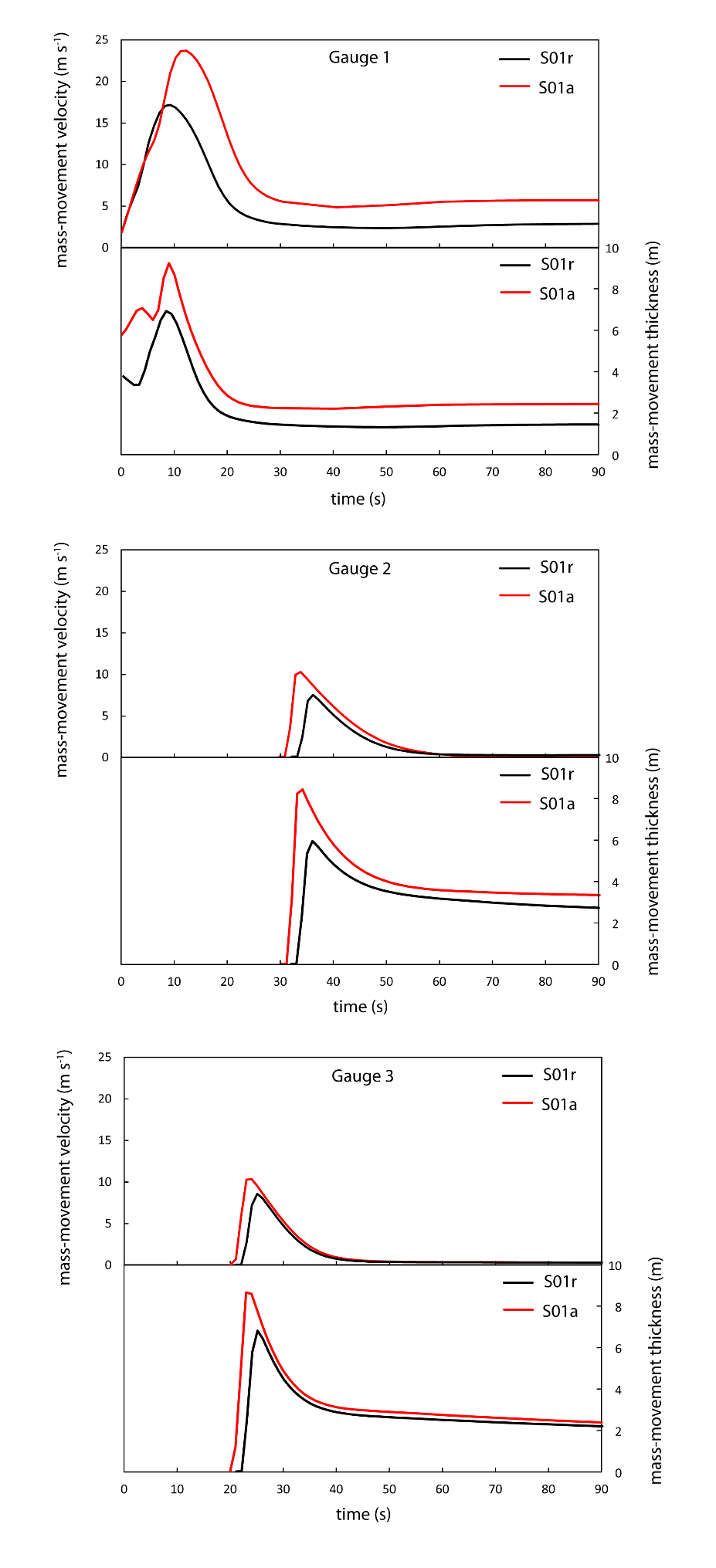
**

**Fig. A5** Time series of simulated mass-movement velocity and thickness at the gauge location (see Fig. A2 for exact position) of the two different numerical simulations S01r and S01a

Table A1: Sediment core locations in Swiss coordinate system LV95

| Core ID | East (m) | North (m) | Water depth (m)  or m a.s.l | Length (cm) | Study |
| --- | --- | --- | --- | --- | --- |
| SIL06-8 | 2’777’946 | 1’145’042 | na | 85 | this study |
| SIL09-1 | 2’777’997 | 1’145’056 | 1798.54 | 515 | this study |
| SIL09-2 | 2’777’979 | 1’145’050 | 1798.25 | 340 | this study |
| SIL09-3 | 2’777’960 | 1’145’045 | 1797.88 | 362 | this study |
| SIL09-4 | 2’777’944 | 1’145’035 | 1797.67 | 394 | this study |
| SIL09-5 | 2’777’926 | 1’145’026 | 1797.52 | 370 | this study |
| SIL09-6 | 2’777’943 | 1’145’076 | 1797.67 | 430 | this study |
| SIL09-7 | 2’777’929 | 1’145’069 | 1797.49 | 227 | this study |
| SIL09-8 | 2’777’920 | 1’145’064 | 1797.96 | 422 | this study |
| SIL09-9 | 2’777’903 | 1’145’057 | 1797.37 | 371 | this study |
| SIL09-10 | 2’777’940 | 1’145’062 | 1797.13 | 455 | this study |
| SIL09-11 | 2’778’025 | 1’145’179 | 1798.15 | 254 | this study |
| SIL09-12 | 2’778’065 | 1’145’204 | 1798.12 | 193 | this study |
| SIL10-1 | 2’777’820 | 1’144’985 | 1.7 | 90 | this study |
| SIL10-2 | 2’777’768 | 1’144’941 | 4.3 | 182 | this study |
| SIL10-3 | 2’777’717 | 1’144’902 | 20.0 | 156 | this study |
| SIL10-4 | 2’777’656 | 1’144’860 | 28.4 | 193 | this study |
| SIL10-5 | 2’777’599 | 1’144’837 | 35.0 | 181 | this study |
| SIL10-6 | 2’777’505 | 1’144’790 | 39.4 | 171 | this study |
| SIL10-7 | 2’777’739 | 1’144’918 | 12.0 | 56 | this study |
| SIL18-1 | 2’777’819 | 1’144’993 | <3 | 50 | this study |
| SIL18-2 | 2’777’808 | 1’145’006 | <3 | 88 | this study |
| SIL18-3 | 2’777’784 | 1’145’021 | <3 | 80 | this study |
| SIL18-4 | 2’777’821 | 1’144’908 | <3 | 105 | this study |
| SIL18-5 | 2’777’840 | 1’144’908 | <3 | 100 | this study |
| SIL18-6 | 2’777’772 | 1’145’067 | <3 | 82 | this study |
| SIL18-7 | 2’777’735 | 1’144’273 | <3 | 67 | this study |
| SIL18-8 | 2’777’878 | 1’144’126 | <3 | 59 | this study |
| SIL18-9 | 2’777’819 | 1’144’135 | <3 | 62 | this study |
| SIL18-10 | 2’777’793 | 1’144’168 | <3 | 51 | this study |
| Sis03-2 | 2’774’670 | 1’142’384 | 27 | 97.5 | Blass et al. (2005) |
| Sis03-22 | 2’777’252 | 1’144’698 | 46 | 84 | Blass et al. (2005) |
| Sis03-23 | 2’777’593 | 1’144’855 | 34 | 78 | Blass et al. (2005) |
| Sis03-28 | 2’777’045 | 1’144’572 | 48 | 92 | Blass et al. (2005) |
| PSS94-2 | 2’777’452 | 1’144’701 | ~70 | 811 | Ohlendorf (1998) |
| PSS94-5 | 2’777’452 | 1’144’702 | ~42 | 283 | Ohlendorf (1998) |

Table A2: Mineralogical composition of sediment samples from core SIL10-1, SIL10-5, and SIL09 in volume percentage

| Core subsample | Composite | Lithological | Qtz | Pl | Kfs | CPx | Mca | Chl | Dol | Cc | Srp | Am | Tlc |
| --- | --- | --- | --- | --- | --- | --- | --- | --- | --- | --- | --- | --- | --- |
|  | depth (cm) | unit | (vol%) | (vol%) | (vol%) | (vol%) | (vol%) | (vol%) | (vol%) | (vol%) | (vol%) | (vol%) | (vol%) |
| SIL10-1-46 | 40 | D (clay cap) | 15 | 9 | 2 | 1 | 38 | 28 | 1 | 1 | 2 | 4 | 0 |
| SIL10-1-51 | 45 | D | 30 | 12 | 3 | 1 | 24 | 22 | 1 | 1 | 1 | 3 | 1 |
| SIL10-1-54 | 48 | E | 12 | 5 | 1 | 1 | 39 | 34 | 3 | 0 | 2 | 4 | 0 |
| SIL10-1-76 | 70 | F | 17 | 5 | 2 | 2 | 33 | 33 | 1 | 0 | 3 | 3 | 0 |
|  |  |  |  |  |  |  |  |  |  |  |  |  |  |
| SIL10-5A-78 | 67 | C | 11 | 5 | 6 | 2 | 26 | 34 | 1 | 2 | 5 | 9 | 0 |
| SIL10-5A-87 | 76 | D (clay cap) | 13 | 7 | 2 | 3 | 29 | 33 | 0 | 1 | 6 | 5 | 0 |
| SIL10-5A-91 | 80 | D | 28 | 17 | 2 | 1 | 27 | 18 | 2 | 1 | 2 | 4 | 0 |
| SIL10-5-98 | 87 | D | 17 | 6 | 1 | 1 | 36 | 36 | 1 | 0 | 0 | 4 | 0 |
| SIL10-5B-20 | 116 | D | 31 | 8 | 0 | 1 | 31 | 22 | 3 | 1 | 1 | 2 | 0 |
| SIL10-5B-70 | 166 | D | 23 | 6 | 1 | 1 | 36 | 27 | 2 | 1 | 2 | 2 | 0 |
|  |  |  |  |  |  |  |  |  |  |  |  |  |  |
|  |  |  |  |  |  |  |  |  |  |  |  |  |  |
| SIL09-4A-44 | 44 | II | 13 | 27 | 4 | 0 | 36 | 15 | 0 | 0 | 1 | 4 | 0 |
| SIL09-4A-49 | 49 | II | 15 | 16 | 2 | 1 | 41 | 20 | 0 | 0 | 1 | 5 | 0 |
| SIL09-4A-52 | 52 | II | 23 | 18 | 1 | 1 | 37 | 14 | 1 | 0 | 1 | 4 | 0 |
| SIL09-4A-60 | 60 | II | 21 | 19 | 1 | 1 | 41 | 13 | 0 | 0 | 1 | 3 | 0 |
| SIL09-4A-85 | 85 | V | 23 | 19 | 1 | 1 | 28 | 22 | 1 | 1 | 1 | 3 | 0 |

Notes: Mineral abbreviations are Qtz = quartz; Pl = plagioclase; Kfs = K-feldspar; CPx = clinopyroxene; Mca = white mica; Chl = chlorite; Dol = dolomite; Cc = calcite; Srp = serpentine; Am = amphibole; and Tlc = talc

Table A3: Mineralogical composition of the sand-sized riverine bedload samples collected at Lake Sils major tributaries in volume percentage

| Riverine bedload sample | Qtz  (vol%) | Pl  (vol%) | Kfs  (vol%) | CPx  (vol%) | Mca  (vol%) | Chl  (vol%) | Dol  (vol%) | Cc  (vol%) | Srp  (vol%) | Am  (vol%) | Tlc  (vol%) |
| --- | --- | --- | --- | --- | --- | --- | --- | --- | --- | --- | --- |
| 1: Aua da Fedoz | 61.02 | 19.46 | 2.52 | 2.61 | 5.06 | 2.06 | 2.78 | 0.00 | 0.00 | 3.66 | 0.83 |
| 2: Lavatera | 84.02 | 5.36 | 1.18 | 1.25 | 1.48 | 2.81 | 0.73 | 0.43 | 1.94 | 0.79 | 0.00 |
| 3: Lavatera + Ova dal Mulin | 87.51 | 5.5 | 0.87 | 1.17 | 1.02 | 1.76 | 0.00 | 0.00 | 1.76 | 0.42 | 0.00 |
| 4: Ova de la Roda | 73.62 | 13.38 | 6.85 | 2.82 | 0.42 | 0.73 | 0.00 | 0.98 | 0.00 | 0.75 | 0.00 |
| 5: Ova dal Crot | 71.63 | 16.1 | 7.99 | 2.22 | 0.65 | 1.07 | 0.00 | 0.00 | 0.00 | 0.34 | 0.00 |
| 6: Fadacla | 74.22 | 11.30 | 4.56 | 1.38 | 2.68 | 1.00 | 4.28 | 0.00 | 0.00 | 0.58 | 0.00 |

Notes: Mineral abbreviations are Qtz = quartz; Pl = plagioclase; Kfs = K-feldspar; CPx = clinopyroxene; Mca = white mica; Chl = chlorite; Dol = dolomite; Cc = calcite; Srp = serpentine; Am = amphibole; and Tlc = talc

Table A4: Elemental concentrations of inorganic and total carbon, total nitrogen, and total sulfur in C sediment samples from core SIL10-1

| Core subsample | Composite | Lithological | IC | CaCO_3_ | TC | TOC | TN | TS | C/N |
| --- | --- | --- | --- | --- | --- | --- | --- | --- | --- |
|  | depth (cm) | units | (wt%) | (wt%) | (wt%) | (wt%) | (wt%) | (wt%) | (mol mol^-1^) |
| SIL10-1-34 | 28 | C | 0 | 0 | 4.08 | 4.08 | 0.347 | 1.449 | 13.7 |
| SIL10-1-39 | 33 | C | 0 | 0 | 3.35 | 3.35 | 0.300 | 1.178 | 13.0 |
| SIL10-1-42 | 36 | C | 0 | 0 | 4.56 | 4.56 | 0.411 | 1.509 | 12.9 |
| SIL10-1-46 | 40 | D (clay cap) | 0 | 0 | 1.58 | 1.58 | 0.091 | 0.194 | 20.2 |
| SIL10-1-48 | 42 | D | 0 | 0 | 0.20 | 0.20 | 0.014 | 0.016 | 16.8 |
| SIL10-1-52 | 46 | D | 0.19 | 1.62 | 1.67 | 1.48 | 0.109 | 0.020 | 15.8 |
| SIL10-1-53 | 47 | E | 0 | 0 | 8.74 | 8.74 | 0.492 | 0.282 | 20.7 |
| SIL10-1-55 | 49 | E | 0 | 0 | 38.75 | 38.75 | 2.221 | 0.894 | 20.4 |
| SIL10-1-59 | 53 | E | 0 | 0 | 23.52 | 23.52 | 1.546 | 0.295 | 17.7 |
| SIL10-1-62 | 56 | F | 0 | 0 | 2.08 | 2.08 | 0.141 | 0.016 | 17.2 |
| SIL10-1-67 | 61 | F | 0 | 0 | 1.11 | 1.11 | 0.076 | 0.007 | 17.0 |
| SIL10-1-72 | 66 | F | 0 | 0 | 1.40 | 1.40 | 0.103 | 0.011 | 15.9 |
| SIL10-1-77 | 71 | F | 0 | 0 | 0.02 | 0.02 | 0.007 | 0 | 3.8 |
| SIL10-1-84 | 78 | F | 0 | 0 | 1.78 | 1.78 | 0.113 | 0 | 18.4 |
| SIL10-1-89 | 83 | F | 0 | 0 | 0.41 | 0.41 | 0.045 | 0.048 | 10.7 |

Notes: IC = inorganic carbon; CaCO_3_ = calcium carbonate; TC = total carbon; TOC = total organic carbon; TN = total nitrogen; TS = total sulfur; and C/N = carbon/nitrogen ratio. TOC concentrations a calculated from the difference between IC and TC concentrations. C/N ratios are calculated from TOC and TN concentrations and are given as weight/weight ratios

Table A5: Elemental concentrations of inorganic and total carbon, total nitrogen, and total sulfur in C sediment samples from core SIL10-5

| Core subsample | Composite | Lithological | IC | CaCO_3_ | TC | TOC | TN | TS | C/N |
| --- | --- | --- | --- | --- | --- | --- | --- | --- | --- |
|  | depth (cm) | units | (wt%) | (wt%) | (wt%) | (wt%) | (wt%) | (wt%) | (mol mol^-1^) |
| SIL10-5A-82 | 71 | C | 0 | 0 | 1.03 | 1.03 | 0.121 | 0.068 | 9.9 |
| SIL10-5A-84 | 73 | C | 0 | 0 | 0.86 | 0.86 | 0.074 | 0.084 | 13.6 |
| SIL10-5A-86 | 75 | C | 0 | 0 | 0.87 | 0.87 | 0.095 | 0.034 | 10.6 |
| SIL10-5A-88 | 77 | D (clay cap) | 0 | 0 | 7.18 | 7.18 | 0.509 | 0.116 | 16.5 |
| SIL10-5A-90 | 79 | D | 0.11 | 0.92 | 0.62 | 0.51 | 0.015 | 0.029 | 39.7 |
| SIL10-5A-98 | 87 | D | 0.03 | 0.28 | 0.73 | 0.70 | 0.067 | 0.019 | 12.2 |
| SIL10-5A-103 | 92 | D | 0.26 | 2.22 | 1.11 | 0.85 | 0.059 | 0.017 | 16.7 |
|  |  |  |  |  |  |  |  |  |  |
| SIL10-5B-10 | 106 | D | 0.29 | 2.42 | 0.76 | 0.47 | 0.016 | 0.041 | 34.3 |
| SIL10-5B-20 | 116 | D | 0.35 | 2.94 | 0.78 | 0.43 | 0.018 | 0.016 | 27.8 |
| SIL10-5B-30 | 126 | D | 0.28 | 2.38 | 0.74 | 0.46 | 0.017 | 0.019 | 31.4 |
| SIL10-5B-60 | 156 | D | 0.19 | 1.60 | 0.67 | 0.48 | 0.018 | 0.030 | 31.0 |
| SIL10-5B-70 | 166 | D | 0.26 | 2.21 | 0.78 | 0.52 | 0.015 | 0.020 | 40.2 |
| SIL10-5B-80 | 176 | D | 0.26 | 2.22 | 0.63 | 0.37 | 0.012 | 0.019 | 36.4 |

Notes: IC = inorganic carbon; CaCO_3_ = calcium carbonate; TC = total carbon; TOC = total organic carbon; TN = total nitrogen; TS = total sulfur; and C/N = carbon/nitrogen ratio. TOC concentrations a calculated from the difference between IC and TC concentrations. C/N ratios are calculated from TOC and TN concentrations and are given as weight/weight ratios

Table A6: Elemental concentrations of inorganic and total carbon, total nitrogen, and total sulfur in C sediment samples from core SIL09-4

| Core subsample | Composite | Lithological | IC | CaCO_3_ | TC | TOC | TN | TS | C/N |
| --- | --- | --- | --- | --- | --- | --- | --- | --- | --- |
|  | depth (cm) | units | (wt%) | (wt%) | (wt%) | (wt%) | (wt%) | (wt%) | (mol mol^-1^) |
| SIL09-4A-0-12-44 | 40 | II | 0 | 0 | 0.30 | 0.30 | 0.036 | 0 | 9.8 |
| SIL09-4A-0-12-49 | 45 | II | 0 | 0 | 0.68 | 0.68 | 0.144 | 0 | 5.5 |
| SIL09-4A-0-12-53 | 49 | II | 0 | 0 | 0.37 | 0.37 | 0.038 | 0 | 11.4 |
| SIL09-4A-0-12-57 | 53 | II | 0 | 0 | 0.46 | 0.46 | 0.033 | 0 | 16.4 |
| SIL09-4A-0-12-61 | 57 | II | 0 | 0 | 0.58 | 0.58 | 0.038 | 0 | 17.7 |
| SIL09-4A-0-12-65 | 61 | D(III) | 0 | 0 | 1.23 | 1.23 | 0.110 | 0.019 | 13.1 |
| SIL09-4A-0-12-68 | 64 | E(IV) | 0 | 0 | 38.63 | 38.63 | 1.707 | 0.972 | 26.4 |
| SIL09-4A-0-12-73 | 69 | E(IV) | 0 | 0 | 42.94 | 42.94 | 1.968 | 1.613 | 25.5 |
| SIL09-4A-0-12-78 | 74 | V | 0 | 0 | 4.34 | 4.34 | 0.275 | 0.276 | 18.4 |
|  |  |  |  |  |  |  |  |  |  |
| SIL09-4B-6-18-33 | 79 | V | 0 | 0 | 1.46 | 1.46 | 0.102 | 0.047 | 16.7 |
| SIL09-4B-6-18-38 | 84 | V | 0 | 0 | 1.42 | 1.42 | 0.296 | 0.025 | 5.6 |
| SIL09-4B-6-18-43 | 89 | VI | 0 | 0 | 0.61 | 0.61 | 0.048 | 0.022 | 14.9 |
|  |  |  |  |  |  |  |  |  |  |
| SIL09-4A-12-24-12 | 94 | VI | 0 | 0 | 0.38 | 0.38 | 0.028 | 0.023 | 15.7 |
| SIL09-4A-12-24-17 | 99 | VI | 0 | 0 | 0.21 | 0.21 | 0.019 | 0.012 | 13.0 |

Notes: IC = inorganic carbon; CaCO_3_ = calcium carbonate; TC = total carbon; TOC = total organic carbon; TN = total nitrogen; TS = total sulfur; and C/N = carbon/nitrogen ratio. TOC concentrations a calculated from the difference between IC and TC concentrations. C/N ratios are calculated from TOC and TN concentrations and are given as weight/weight ratios
